# Oligomeric amyloid-β targeted contrast agent for MRI evaluation of Alzheimer’s disease mouse models

**DOI:** 10.3389/fphar.2024.1392729

**Published:** 2024-06-04

**Authors:** Jang Woo Park, Yunan Tian, Sang-Tae Kim, Chanwoo Park, Yu Mi Kim, Hye Kyung Chung, Kyeong Min Kim, Geon-Ho Jahng

**Affiliations:** ^1^ Korea Radioisotope Center for Pharmaceuticals, Korea Institute of Radiological and Medical Sciences, Seoul, Republic of Korea; ^2^ Department of Medicine, Graduate School, Kyung Hee University College of Medicine, Seoul, Republic of Korea; ^3^ J&Pharma, Neuroscience Research Institute, Healthcare Innovation Park, Seongnam City, Republic of Korea; ^4^ Research Institute of Radiological and Medical Sciences, Korea Institute of Radiological and Medical Sciences, Seoul, Republic of Korea; ^5^ Radiological and Medico Oncological Sciences, University of Science and Technology (UST), Seoul, Republic of Korea; ^6^ Department of Radiology, Kyung Hee University Hospital at Gangdong, College of Medicine, Kyung Hee University, Seoul, Republic of Korea

**Keywords:** MRI contrast agent, DNA aptamer, oligomeric amyloid-beta, ApoE mouse model, tau mouse model, time-dependent signal enhancement

## Abstract

**Background:**

Oligomeric amyloid beta (oAβ) is a toxic factor that acts in the early stage of Alzheimer’s disease (AD) and may initiate the pathologic cascade. Therefore, detecting oAβ has a crucial role in the early diagnosis, monitoring, and treatment of AD.

**Purpose:**

The purpose of this study was to evaluate MRI signal changes in different mouse models and the time-dependent signal changes using our novel gadolinium (Gd)-dodecane tetraacetic acid (DOTA)- ob5 aptamer contrast agent.

**Methods:**

We developed an MRI contrast agent by conjugating Gd-DOTA-DNA aptamer called ob5 to evaluate its ability to detect oAβ deposits in the brain using MRI. A total of 10 control mice, 9 3xTg AD mice, and 11 APP/PS/Tau AD mice were included in this study, with the age of each model being 16 or 36 weeks. A T1-weighted image was acquired at the time points before (0 min) and after injection of the contrast agent at 5, 10, 15, 20, and 25 min. The analyses were performed to compare MRI signal differences among the three groups and the time-dependent signal differences in different mouse models.

**Results:**

Both 3xTg AD and APP/PS/Tau AD mouse models had higher signal enhancement than control mice at all scan-time points after injection of our contrast media, especially in bilateral hippocampal areas. In particular, all Tg AD mouse models aged 16 weeks showed a higher contrast enhancement than those aged 36 weeks. For 3xTg AD and APP/PS/Tau AD groups, the signal enhancement was significantly different among the five time points (0 min, 5 min, 10 min, 15 min, 20 min, and 25 min) in multiple ROI areas, typically in the bilateral hippocampus, left thalamus, and left amygdala.

**Conclusion:**

The findings of this study suggest that the expression of the contrast agent in different AD models demonstrates its translational flexibility across different species. The signal enhancement peaked around 15–20 min after injection of the contrast agent. Therefore, our novel contrast agent targeting oAβ has the potential ability to diagnose early AD and monitor the progression of AD.

## Introduction

### Histopathologic hallmarks of Alzheimer’s disease (AD)

Alzheimer’s disease (AD) is a neurodegenerative disorder characterized by extracellular amyloid plaques, intracellular tau tangles, and neuronal loss. Developing imaging tools for these hallmarks is important for the diagnosis and treatment of AD. Some imaging markers are currently used in clinics, such as amyloid plaque positron emission tomography (PET) radiotracers, tau protein PET radiotracers, and magnetic resonance imaging (MRI) of hippocampal atrophy, especially in the late stage of the disease. However, amyloid-beta (Aβ) has several forms, such as non-toxic monomeric Aβ (mAβ) and toxic oligomeric Aβ (oAβ), and the misfolding of mAβ precedes the formation of oligomers (Huang and Liu, 2020).

### Oligomeric amyloid-beta (oAβ) imaging

Oligomeric forms of amyloid precursor protein (APP) cleavage products play a pivotal role in the early stages of AD (Thomas et al., 2016; Chae et al., 2020). To facilitate the early diagnosis of AD patients, it is essential to detect oAβ rather than amyloid plaques. oAβ imaging tool because oAβ is considered to be the most toxic and pathogenic form of Aβ in AD, as it can impair synaptic function, induce neuroinflammation, and trigger neuronal death, leading to cognitive decline and dementia (Viola et al., 2022; Pyun et al., 2023). However, current imaging tools for AD diagnosis mainly target amyloid plaques, which are aggregates of fibrillar Aβ that accumulate in the brain. Amyloid plaques do not correlate well with disease progression and are not present at the earliest stages of the disease. Therefore, imaging tools that can detect oAβ *in vivo* would be more useful for early diagnosis, monitoring, and treatment of AD (Viola et al., 2022; Pyun et al., 2023). Recently, we developed an MRI contrast agent to detect oAβ based on the conjugation of gadolinium (Gd)-dodecane tetraacetic acid (DOTA) to a DNA aptamer called ob5 which can bind to a given target with high affinities (Kim et al., 2023). We demonstrated the ability of a Gd-DOTA-ob5- cyanine5.5 (cy5.5) contrast agent to detect oAβ using fluorescence imaging in cell experiments, and *ex-vivo* and *in-vivo* experiments using APP/PS/ApoE knockdown (KD) AD mouse model, also known as 3xTg AD mice (Kim et al., 2023).

### Mouse models to represent humanoid Alzheimer’s disease

To study the effects of oAβ on the brain, some researchers have developed mouse models that represent oAβ deposits and toxicity. Different types of mouse models can be used to mimic oAβ pathology, depending on the methods and targets of genetic engineering or treatment. The APP/PS AD mouse is a well-known model that has oAβ from more than 10 weeks of age, as well as amyloid plaques in the brain at later stages. The 3xTg AD mouse, which is APP/PS/ApoE knockout (KO), is the model that is often used. Another mouse model is APP/PS/Tau AD. Both these mouse models have been used to evaluate oAβ deposits in the brain.

Whenever a novel MRI contrast agent is developed, it is important to evaluate the MRI signal changes in different mouse models and the time-dependent signal changes after injection of the contrast agent. Therefore, the objectives of this study were 1) to evaluate the signal enhancement using our developed Gd-DOTA-ob5 aptamer MRI contrast agent in 3xTg, APP/PS/Tau, and control C57BL/6 mouse models for early diagnosis of AD using MRI and 2) to evaluate the time-dependent signal changes after injection of the contrast agent.

## Materials and methods

### Novel contrast agent for targeting oligomer amyloid-beta

We developed a novel MRI contrast agent by conjugating a Gd-DOTA-DNA aptamer, named ob5, which was constructed by amide bond formation, for the detection of oAβ deposits in the brain (Jahng and Kim, 2017; Kim et al., 2023). We used the Selective Evolution of Ligands by Exponential enrichment (SELEX) method (Burke and Gold, 1997; Horii et al., 2010) to prepare selective targeting nucleotides that showed high binding affinity to the oAβ conformation. After preparing Gd-DOTA as previously described (Kielar et al., 2010; Huynh et al., 2017), we synthesized Gd-DOTA-ob5 as described previously (So et al., 2006; Dudeffant et al., 2017; Aminololama-Shakeri et al., 2019; Li and Meade, 2019; Kim et al., 2023) and evaluated its ability to detect oAβ deposits in the brain using MRI.

## Animals

We purchased AD model mice of 3xTg AD and APP/PS/Tau AD from JN Pharma company (Seongnam, Kyunggeedo, Republic of Korea) and the control mice of C57BL/6 non-transgenic (non-Tg) mice from Orient Bio (Seongnam, Kyunggeedo, Republic of Korea). The animal experiments were approved by the Institutional Animal Care and Use Committee of Korea Radioisotope Center for Pharmaceuticals (Seoul, Republic of Korea) (kirams 2023-0064). All mice were housed under controlled conditions of a 12-h light/dark cycle at 21°C ± 2 °C and 50% ± 10% humidity.

Table 1 summarizes the demographic information of the number of animals, ages, and concentration of our Gd-DOTA-ob5 contrast agent. In this study, we used ten control, nine 3xTg AD, and eleven APP/PS/Tau AD mice. We used mice aged 16 and 36 weeks for each model. Age was not significantly different among the three groups tested by the Kruskal–Wallis test (F = 0.340, *p* = 0.796). All mice were male. We used the two different levels of concentrations of our contrast agent for each model. For the low concentration, we injected 0.4948 μL per g of the mouse for 3xTg, 0.4929 μL per g of the mouse for APP/PS/Tau, and 0.6349 μL per g of the mouse for control. For the high concentration, we injected 1.99268 μL per g of the mouse for 3xTg, 1.984 μL per g of the mouse for APP/PS/Tau, and 2.576 μL per g of the mouse for control. The concentration was not significantly different among the three groups tested by the chi-squared test (χ^2^ = 0.091, *p* = 0.763).

**TABLE 1:** Demographics and concentration of contrast agent.

Group	Control (1)	3xTg (2)	APP/PS/Tau (3)	*p*-value (*post hoc*)
participants	10	9	11	30 (total)
^a^Age (weeks)	^b^6 for16 weeks	^b^4 for16 weeks	^b^6 for16 weeks	F = 0.340, *p* = 0.796
	^b^4 for 36 weeks	^b^5 for 36 weeks	^b^5 for 36 weeks	
^b^Concentration (high/low)	3/7 (30.0%/70.0%)	4/5 (55.6%/44.4%)	5/6 (45.5%/54.5%)	χ^2^ = 0.029, *p* = 0.866
				χ^2^ = 0.200, *p* = 0.655
				χ^2^ = 0.091, *p* = 0.763

Data of age are presented as median (range).

^a^ The group difference of age was tested by Kruskal–Wallis test.

^b^ The group difference of concentration of contrast agent was tested by the Chi-squared test.

### MRI experiments

All MRI experiments were performed on a 9.4 T animal MRI scanner (94/20 USR, Bruker, Ettlingen, Germany). A quadrature birdcage RF resonator with an inner diameter of 40 mm was used for signal transmission and reception. To acquire *in vivo* MR images using our Gd-DOTA-ob5 contrast agent, animals were anesthetized with 1.5%–2.0% inhalational isoflurane in oxygen. Animals were subjected to respiratory gating at a rate of 30–50 breaths/min (SA Instruments, Stony Brook, NY, United States) without monitoring cardiovascular gating.

To evaluate MRI signal changes in the whole brain and specific brain areas before and after intravenous injection in the tail vein of the Gd-DOTA-ob5 contrast agent, T1-weighted images were acquired before and after injecting our contrast agent. After injection of the contrast agent, T1-weighted images were repeatedly scanned at approximately 5-minute intervals until 25 min after injection. T1-weighted images were obtained by a rapid acquisition with relaxation enhancement (RARE) spin-echo pulse sequence with the following parameters: TR = 467.7 ms, TE = 6.7 ms, FOV = 20 × 20 mm^2^, NSA = 3, RARE factor = 1, frequency direction = left-right, pixel BW = 0.4807 kHz, matrix size = 200 × 200, number of slices = 30 with slice thickness = 0.7 mm, and voxel size = 0.10 × 0.10 × 0.7 mm^3^. The scan time for each measurement was 4 min 40 s.

Furthermore, T2-weighted images were also acquired with a TurboRARE pulse sequence before and after injecting the Gd-DOTA-ob5 contrast agent with the same imaging parameters as T1-weighted images except the following parameters: TR = 2,500 ms, TE = 25 ms, NSA = 4, RARE factor = 8, pixel BW = 0.3367 kHz, matrix size = 128 × 128, and voxel size = 0.156 × 0.156 × 0.7 mm^3^. The scan time for each measurement was 2 min 40 s. The T2-weighted images after the contrast injection were acquired at the end of each experiment for each animal.

### Processing of MRI data

DICOM images were converted into the NIFTI image format using PMOD software (version 3.8, PMOD Group, Graubünden, Switzerland). We preprocessed NIFITI images using the Statistical Parametric Mapping (SPM12) software (Wellcome Trust Centre for Neuroimaging, UCL Institute of Neurology, London, United Kingdom; www.fil.ion.ucl.ac.uk/spm) according to the following steps. First, all the images were magnified by 10 for the SPM Display function. Second, T1-weighted images after contrast injection were co-registered with those before the injection of the contrast agent (CA). Third, the T2-weighted image after contrast injection was co-registered with those before the injection of the CA. Fourth, T2-weighted images were co-registered with T1-weighted images before the injection of the CA. Fifth, the T2-weighted image and other images were spatially normalized into the C57BL/6 mouse brain template (Hikishima et al., 2017) Finally, the signal intensity of the post-contrast T1-weighted images was adjusted in each voxel by pre-contrast enhanced images T1-weighted image the following equation as S(t)=(Spost-Spre)*100/Spre, where Spost is the signal intensity at post = 5, 10, 15, 20, and 25 min after injection of contrast agent and Spre is the signal intensity at before contrast injection. We drew noise areas in the image before the injection of the CA for each mouse by MRIcro software. Then, the signal-noise ratio (SNR) of all T1-weighted images was calculated by the following equation: SNR = SI*100/Smean noise, where SI is the signal intensity at each scan-time point and Smean noise is the mean value of its noise.

### Confocal microscopy analysis

The details of the immunofluorescence imaging to image oAβ deposits were described in our previous paper (Kim et al., 2023). Briefly, mice were sacrificed and perfused with 0.9% saline. The brains were removed and post-fixed with 4% paraformaldehyde (PFA) in 0.1 M phosphate buffer saline (PBS: pH 7.4) at 4°C overnight. The brains were cryo-sectioned into 25 µm-thick sections with a cryostat microtome (Leica CM 1950; Leica Instruments, Nussloch, Germany). Cryosections (25 µm) were mounted on Superfrost Plus slides (Thermo Scientific), fixed for 10 min with 4% PFA in 30% sucrose in PBS, washed, and blocked with 10% BSA (bovine serum albumin) + 0.04% Triton X-100 in 0.05 M PBS for 1 h. Free-floating sections were incubated with 4% bovine serum albumin in PBS for 1 h, then reacted with monoclonal anti-β-amyloid antibody (Bam10)-QD525 and ob5 aptamer-conjugated QD565 at 4°C overnight. The monoclonal antibody BAM-10 (MA1-91209) (ThermoFisher, Waltham, United States) recognizes the epitope for the N-terminus (1–12 amino acid residues) of Aβ1-42 for amyloid plaques in the brains of AD mouse models (Lee et al., 2009). Confocal images were acquired on a spinning disk confocal imaging system (LSM710; Carl Zeiss AG, Oberkochen, Germany).

### Statistical analyses

#### Voxel-based analyses

All the maps were smoothed with an 8 × 8 × 8 mm^3^ full-width half-maximum Gaussian kernel. We performed the following voxel-based analyses. First, the SNR was compared among the three mice groups using ANOVA for each scan time point (pre, 5, 10, 15, 20, and 25 min each). Second, the SNR signal changes were compared among the six scan time points using paired t-tests (pre, 5, 10, 15, 20, and 25 min each) with the concentration of the contrast agent as the covariate. Because the number of animals was small in each group, we did not use age as a covariate during the voxel-based analyses. Third, the group difference of SNR between 16-week-old and 36-week-old Tg AD mice using both Tg AD mouse models was compared for each scan-time point using a two-sample t-test with the mice model as the covariate. Furthermore, the group difference of SNR between Tg AD mouse models and the control mouse group of the same age was compared for each scan-time point using the two-sample t-test. The significance level was α = 0.005 with the threshold of 50 contiguous voxels without correcting for multiple comparisons. The voxel-based analyses were performed to select brain areas for the region-of-interest (ROI)-based analyses.

#### ROI-based analyses

**FIGURE 1:**
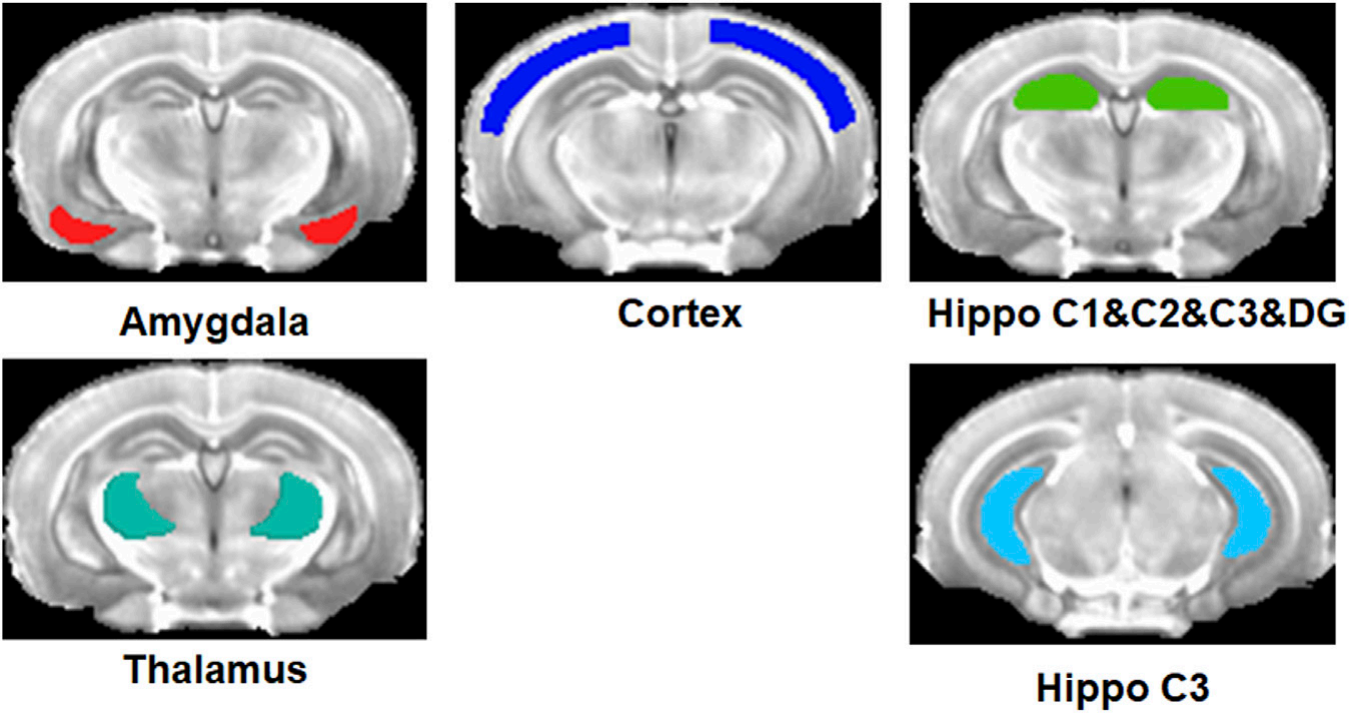
Regions-of-interest (ROIs) defined at the right and left amygdala, cortex, thalami, and frontal part and posterior part of the hippocampus.

ROIs were defined at the right and left frontal part of the hippocampus and posterior part of the hippocampus, separately, cortex, amygdala, and thalami as shown in Figure 1. The regions were primarily referenced to Mouse Coronal Atlas (Reference Atlas: Allen Brain Atlas: Mouse Brain (brain-map.org)) and mapped by MRIcro software. The values of the signal intensity of the post-contrast T1-weighted images in each ROI were obtained using Marsbar software (Matthew Brett, http://marsbar. sourceforge.net). We performed the following analyses using the ROI data. First, the signal intensity was compared between left and right for each ROI for each animal model and each scan point using the Wilcoxon signed-rank test. Second, the three group comparisons of the signal intensity were performed using the Kruskal–Wallis test for 5, 10, 15, 20, and 25 min in each ROI. Third, the signal change between scan time points was tested using the Friedman test for each mouse model. Fourth, the group difference of SNR between 16-week-old and 36-week-old Tg AD mice using both Tg AD mouse models was compared for each scan-time point using a two-sample t-test with the mice model as the covariate. Furthermore, the group difference of SNR between Tg AD mouse models and the control mouse group of the same age was compared for each scan-time point using the two-sample t-test. Fifth, the Mann-Whitney test was performed to compare group differences in the signal intensity between the control and AD mouse models in low concentrations of the proposed contrast agent at each scan time point in each ROI. We did not perform this analysis with the high concentrations of the contrast agent because the number of control mice with high contrast was only 3. Moreover, the Mann-Whitney test was also used to compare signal intensities between high and low concentrations in only Tg AD mouse models at each scan-time point in each ROI. The significance level of α = 0.05 was used. MedCalc statistical software (http://www.medcalc.org/, Ostend, Belgium) was used to analyze the ROI data.

## Results

### Voxel-based analyses

#### Group comparison between mouse models for each scan-time point

**FIGURE 2:**
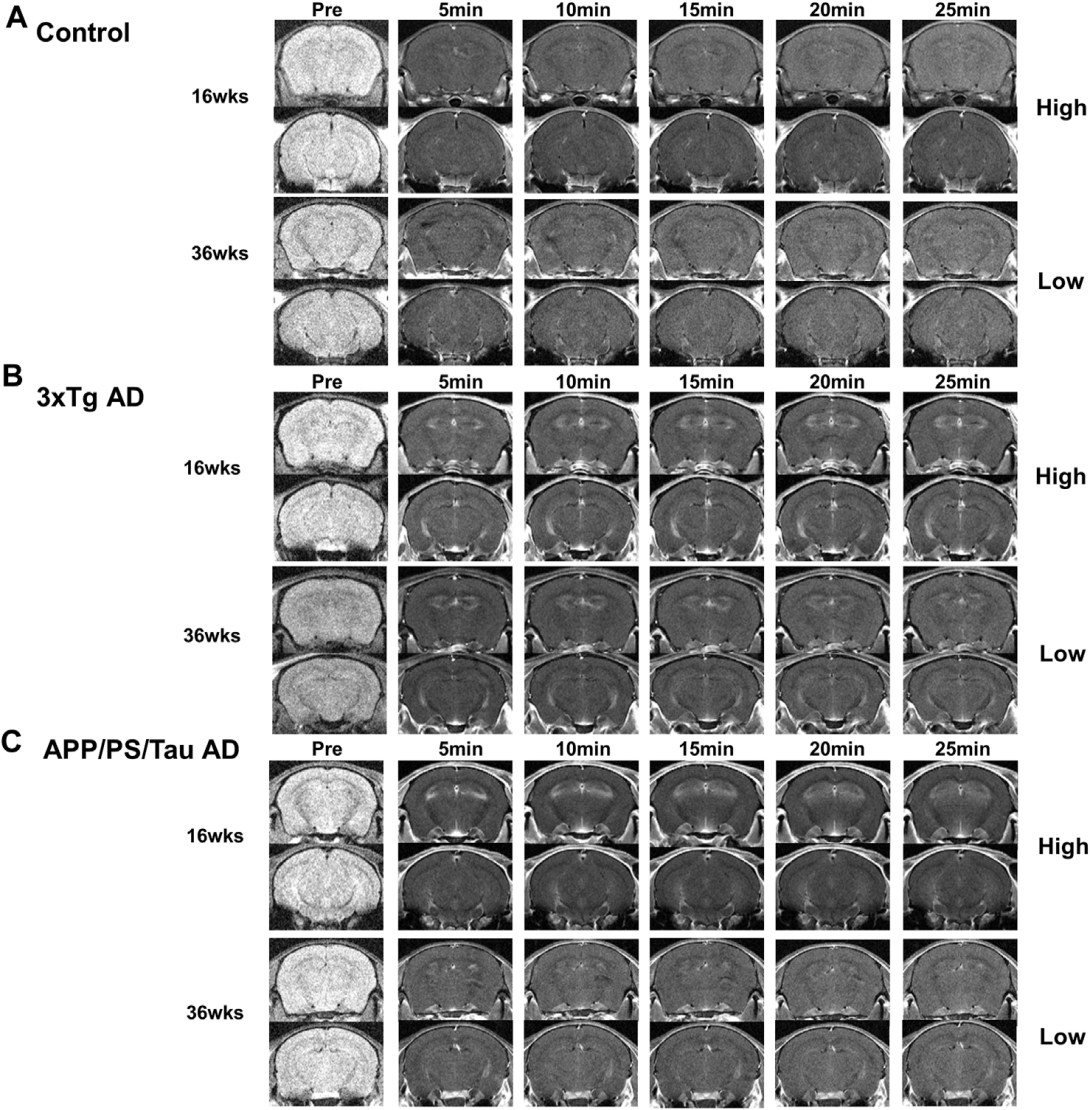
Representative images obtained from Control **(A)**, 3xTg AD **(B)**, and APP/PS/Tau AD **(C)** mice before and after injection of our proposed contrast agent. For both 3xTg AD (B) and APP/PS/Tau AD (C) mice, T1-weighted images showed a signal increase with our contrast agent from 5 min to 25 min after injection of the contrast agent. Signal enhancement looks higher with 16-week-old mice than with 36-week-old mice for both models.

**FIGURE 3:**
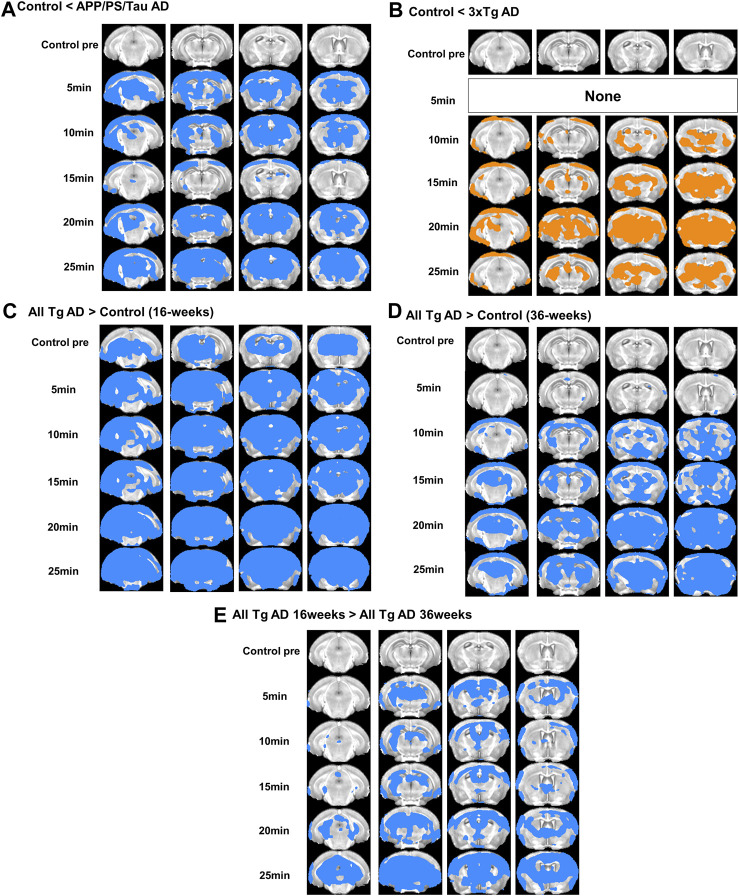
Result of the voxel-based comparison of the signal enhancement between the mouse groups in 5, 10, 15, 20, and 25 min after injection of our proposed contrast agent. Results show comparison between APP/PS/Tau AD and control groups **(A)**, between 3xTg AD and control groups **(B)**, between 16 weeks-aged Tg AD and 16 weeks-aged control mice **(C)**, and between 36 weeks-aged Tg AD and 36 weeks-aged control mice **(D)**, and between 16 weeks-aged Tg AD and 36 weeks-aged Tg AD mice **(E)**.

Figure 2 shows the representative images obtained from the three different mouse models before and after injection of our proposed contrast agent. For the control mice, signals were not strongly enhanced after injection of our contrast agent for both 16 and 36-week-old mice. However, for both 3xTg AD and APP/PS/Tau AD mice, T1-weighted images showed a signal increase after injection of our contrast agent. Figure 3 shows the result of the voxel-based comparison of the signal enhancement between the different mouse groups in 5, 10, 15, 20, and 25 min after injection of our proposed contrast agent. The APP/PS/Tau AD mice (Figure 3A) had higher signal enhancement than control mice at all scan-time points after injection of our contrast media in large brain areas. The 3xTg AD mice (Figure 3B) also had higher signal enhancement than control mice at all scan-time points, except at 5 min, after injection of our contrast media. The signal enhancement was not statistically significant between 3xTg AD mice and APP/PS/Tau AD mice at each scan-time point after injection of our contrast media. The result of the comparison between 16-week-old Tg AD and 16-week-old control mice (Figure 3C) or between 36-week-old Tg AD and 36-week-old control mice (Figure 3D) showed that for each scan-time point, signal enhancements in 16-week-old Tg mice were higher than in 16-week-old control mice (Figure 3C) and signal enhancements in 36-week-old Tg mice were higher than in 36-week-old control mice (Figure 3D). Furthermore, signal enhancements were higher in 16-week-old Tg mice than in 36-week-old Tg mice for each scan-time point (Figure 3E).

#### Comparison between scan-time points for each mouse model

**FIGURE 4:**
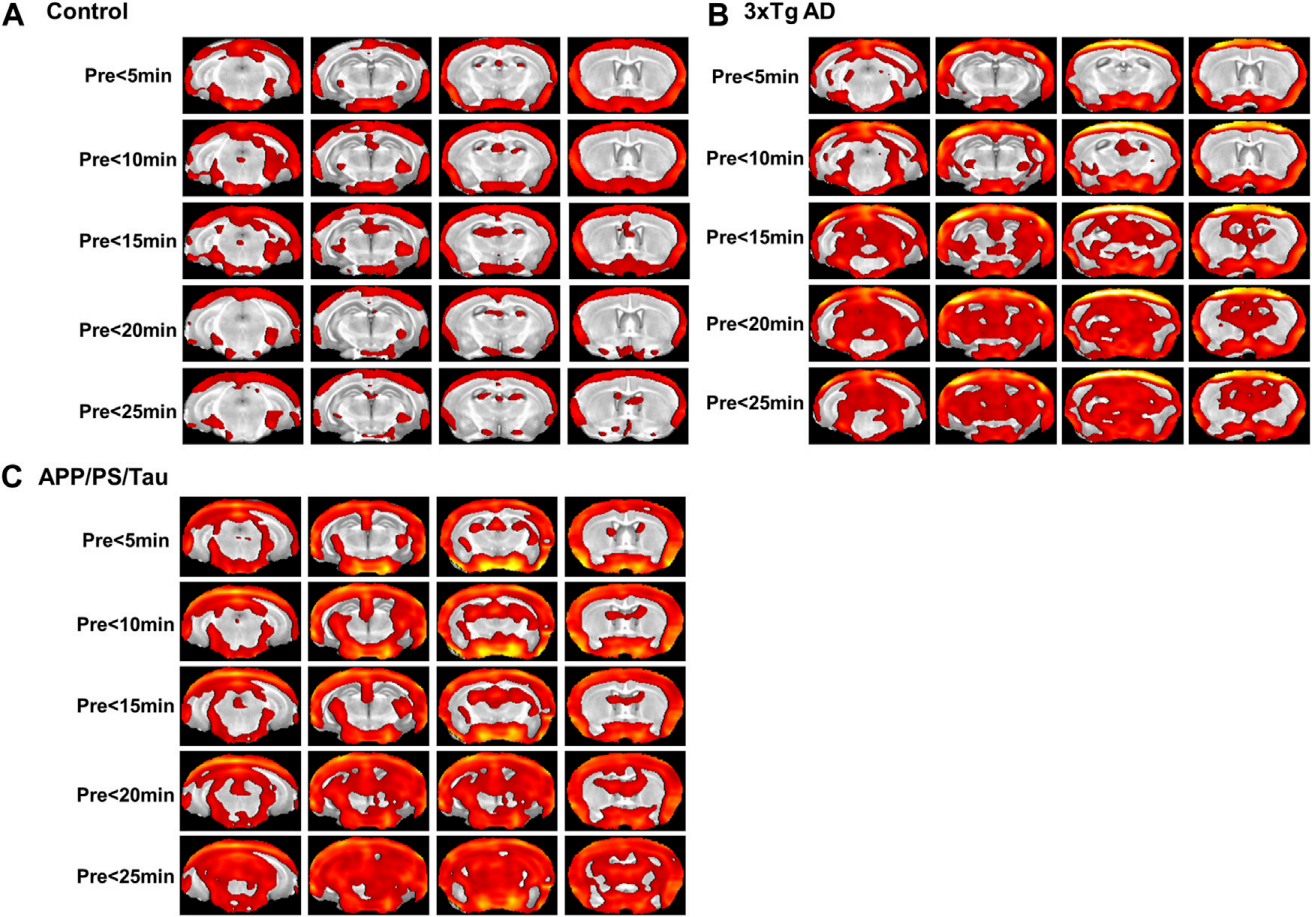
Result of the voxel-based comparison of the signal enhancement between before (pre) and after injection of the proposed contrast agent for the control mouse model group **(A)**, 3xTg AD model group **(B)**, and APP/PS/Tau model group **(C)**. No significant differences were found when signal intensity was compared between other scan-time points after injection of the proposed contrast agent for each mouse model group.

For the 3xTg AD and APP/PS/Tau AD mice, Figure 2 shows that MRI signals increased in 5 min and remained elevated until 25 min after injection of the contrast agent. Signal enhancement appeared higher with 16-week-old mice than with 36-week-old mice for both models. Figure 4 shows that signal enhancement was significantly different before the injection of the contrast agent and other time points after the injection of the contrast agent for both 3xTg AD and APP/PS/Tau AD models. However, there were no significant differences in signal enhancement between scan-time points after the injection of contrast agent for both 3xTg AD and APP/PS/Tau AD models.

### ROI-based analyses

#### Group comparison between mouse models for each scan-time point

**FIGURE 5:**
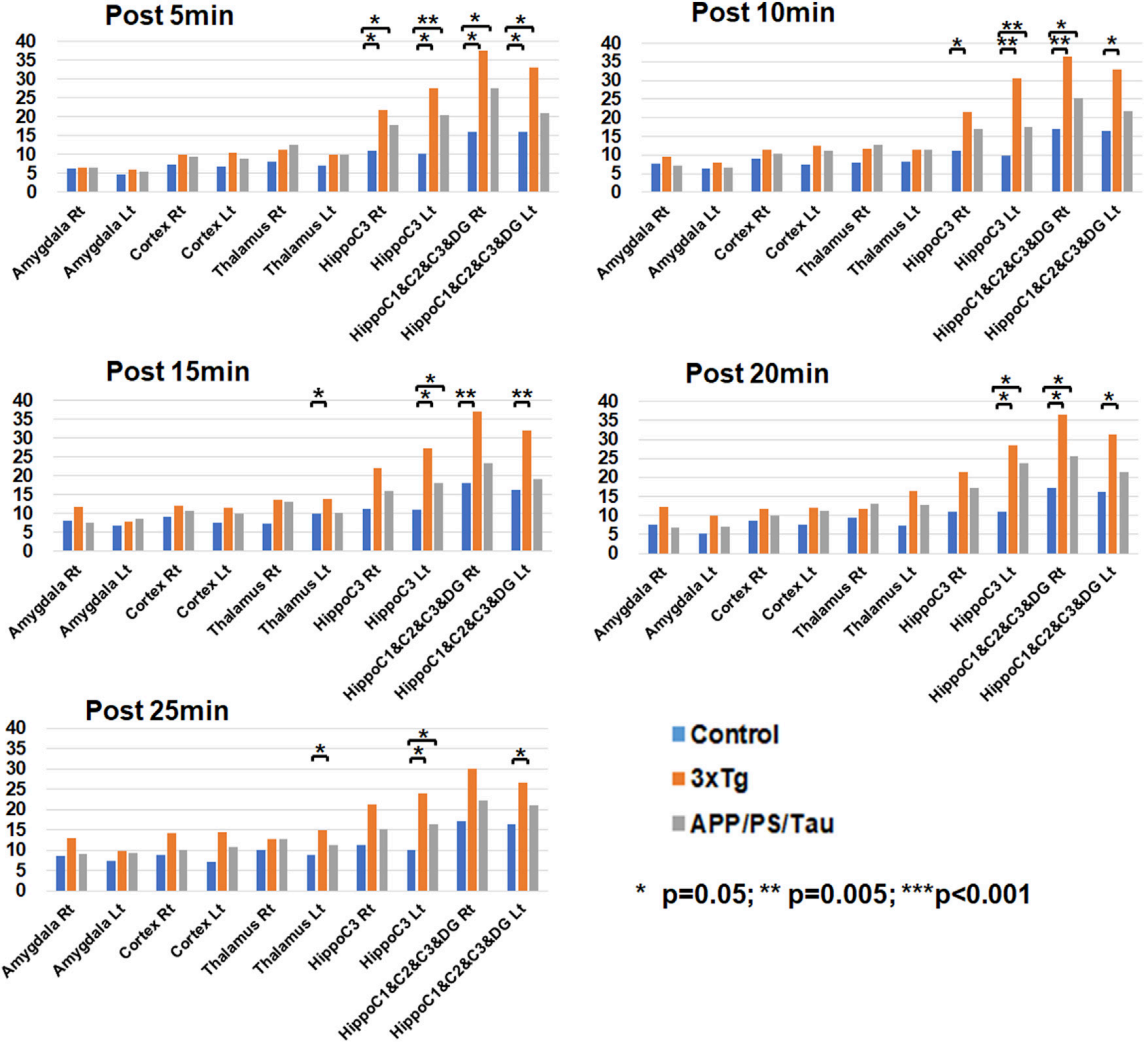
Result of region-of-interest (ROI)-based comparison of the signal enhancement between the three mouse groups for each scan-time point. Data list in Supplementary Table S1 as the median (25th −75th percentile) of the signal enhancement at the scanned times of 5, 10, 15, 20, and 25 min after injection of the contrast media.

Figure 5 shows the result of the ROI-based comparison of the signal enhancement between the three mouse groups for each scan-time point. Supplementary Table S1 also lists the median (25th −75th percentile) of the signal enhancement at the scanned times of 5, 10, 15, 20, and 25 min after injection of the contrast media and the result of the group differences of the signal enhancement between the three different mouse models for each scan-time point.

At the 5 min scan after injection of our contrast agent, the 3xTg mouse group had significantly higher signal enhancement than the control mouse group at the left (*p* = 0.010) and right (*p* = 0.011) hippocampus C1&C2&C3&DG and the left (*p* < 0.001) and right (*p* = 0.006) hippocampus C3 areas. In addition, the APP/PS/Tau mouse group had significantly higher signal enhancement than the control mouse group at the left (*p* = 0.041) and right (*p* = 0.011) hippocampus C1&C2&C3&DG and the left (*p* = 0.003) and right (*p* = 0.041) hippocampus C3 areas.

At the 10 min scan after injection of our contrast agent, the 3xTg mouse group had significantly higher signal enhancement than the control mouse group at the left (*p* = 0.011) and right (*p* = 0.002) hippocampus C1&C2&C3&DG and the left (*p* = 0.003) and right (*p* = 0.018) hippocampus C3 areas. In addition, the APP/PS/Tau mouse group had significantly higher signal enhancement than the control mouse group at the right hippocampus C1&C2&C3&DG (*p* = 0.035) and the left hippocampus C3 (*p* = 0.005) areas.

At the 15 min scan after injection of our contrast agent, the 3xTg mouse group had significantly higher signal enhancement than the control mouse group at the left (*p* = 0.003) and right (*p* = 0.018) hippocampus C1&C2&C3&DG, the left hippocampus C3 (*p* = 0.007), and the left thalamus (*p* = 0.011) areas. In addition, the APP/PS/Tau mouse group had significantly higher signal enhancement than the control mouse group at the left hippocampus C3 (*p* = 0.024) area.

At the 20 min scan after injection of our contrast agent, the 3xTg mouse group had significantly higher signal enhancement than the control mouse group at the left (*p* = 0.014) and right (*p* = 0.009) hippocampus C1&C2&C3&DG and the left hippocampus C3 (*p* = 0.018) areas. In addition, the APP/PS/Tau mouse group had significantly higher signal enhancement than the control mouse group in the right hippocampus C1&C2&C3&DG (*p* = 0.029) and the left hippocampus C3 (*p* = 0.018) areas.

At the 25 min scan after injection of our contrast agent, the 3xTg mouse group had significantly higher signal enhancement than the control mouse group at the left hippocampus C1&C2&C3&DG (*p* = 0.014), the left hippocampus C3 (*p* = 0.022), and the left thalamus (*p* = 0.009) areas. In addition, the APP/PS/Tau mouse group had significantly higher signal enhancement than the control mouse group at the left hippocampus C3 (*p* = 0.017) area.

Supplementary Table S2 lists the results of the comparison of the signal enhancement for each scan-time point between 16- and 36-week-old mice using both Tg mouse models. Supplementary Table S3 lists the results of comparing the signal enhancement for each scan-time point between 16-week-old control and 16-week-old Tg mouse models. Supplementary Table S4 lists the results of comparing the signal enhancement for each scan-time point between 36-week-old control and 36-week-old Tg mouse models.

#### Comparison between control and AD mouse models for the low concentration of the proposed contrast agent

Figure 2 shows signals were significantly enhanced at high concentrations compared to low concentrations in both 3xTg AD and APP/PS/Tau AD mice. Table 2 shows the results of the ROI-based comparison of signal changes in each region-of-interest (ROI) brain area between control and AD model mice for the low concentration of the proposed contrast agent at each time point. For the low concentration, signals were significantly different between the control and AD Tg mouse groups in the hippocampus C1&C2&C3&DG at 5 min, 10 min, 15 min, and 20 min. Furthermore, signals were significantly different between the control and AD Tg mouse groups in the hippocampus C3 at 5 min, 10 min, and 15 min. However, signals were not significantly different between the control and AD mouse model: 1) in the defined hippocampus C3 ROI at 20 min, 2) in all defined ROIs at 25 min, and 3) in other defined ROIs of the amygdala, thalamus, and cortex at all scanned time points.

**TABLE 2:** Comparison of signal changes in each region-of-interest (ROI) brain area between control and AD model mice for the low concentration of the proposed contrast agent.

Scan time (min)	Concentration	ROI	Control (N = 7)	AD model (N = 11)	*p*-value
5	Low	HippoC1&C2C3&DG	12.203 (7.182–25.677)	23.219 (19.385–38.948)	0.021
	Low	HippoC3	9.393 (6.653–15.741)	19.499 (16.185–32.453)	0.004
10	Low	HippoC1&C2C3&DG	16.722 (4.040–21.692)	21.539 (16.960–37.546)	0.021
	Low	HippoC3	9.208 (4.917–13.844)	18.384 (13.322–37.410)	0.016
15	Low	HippoC1&C2C3&DG	16.703 (3.024–20.278)	21.482 (17.561–36.729)	0.027
	Low	HippoC3	10.774 (4.750–13.867)	17.562 (11.282–38.235)	0.033
20	Low	HippoC1&C2C3&DG	17.343 (2.372–19.179)	22.052 (16.326–35.264)	0.042
	Low	HippoC3	10.901 (4.023–14.757)	16.673 (10.904–34.544)	0.063

Data list the median (95% confidence interval of median) value for the control and AD, mouse models experimented with a low concentration of the proposed contrast agent.

For the injection of the low concentration of the proposed contrast agent, signals were not significantly different between the control (N = 7) and AD, mouse model (N = 11): 1) in the defined hippocampus C3 ROI, at 20 min, 2) in all defined ROIs, at 25 min, and 3) in the amygdala, thalamus, and cortex ROIs, at all scanned time points.

For the injection of the high concentration of the proposed contrast agent, signals were not significantly different between the control (N = 3) and AD, mouse model (N = 9) in all defined ROIs, at each time point.

For the injection of the high concentration of the proposed contrast agent, signals were not significantly different between the control (N = 3) and AD mouse model (N = 9) in all defined ROIs at each time point. This may be related to too much small population for the control mice.

#### Comparison between scan-time points for each mouse model

**FIGURE 6:**
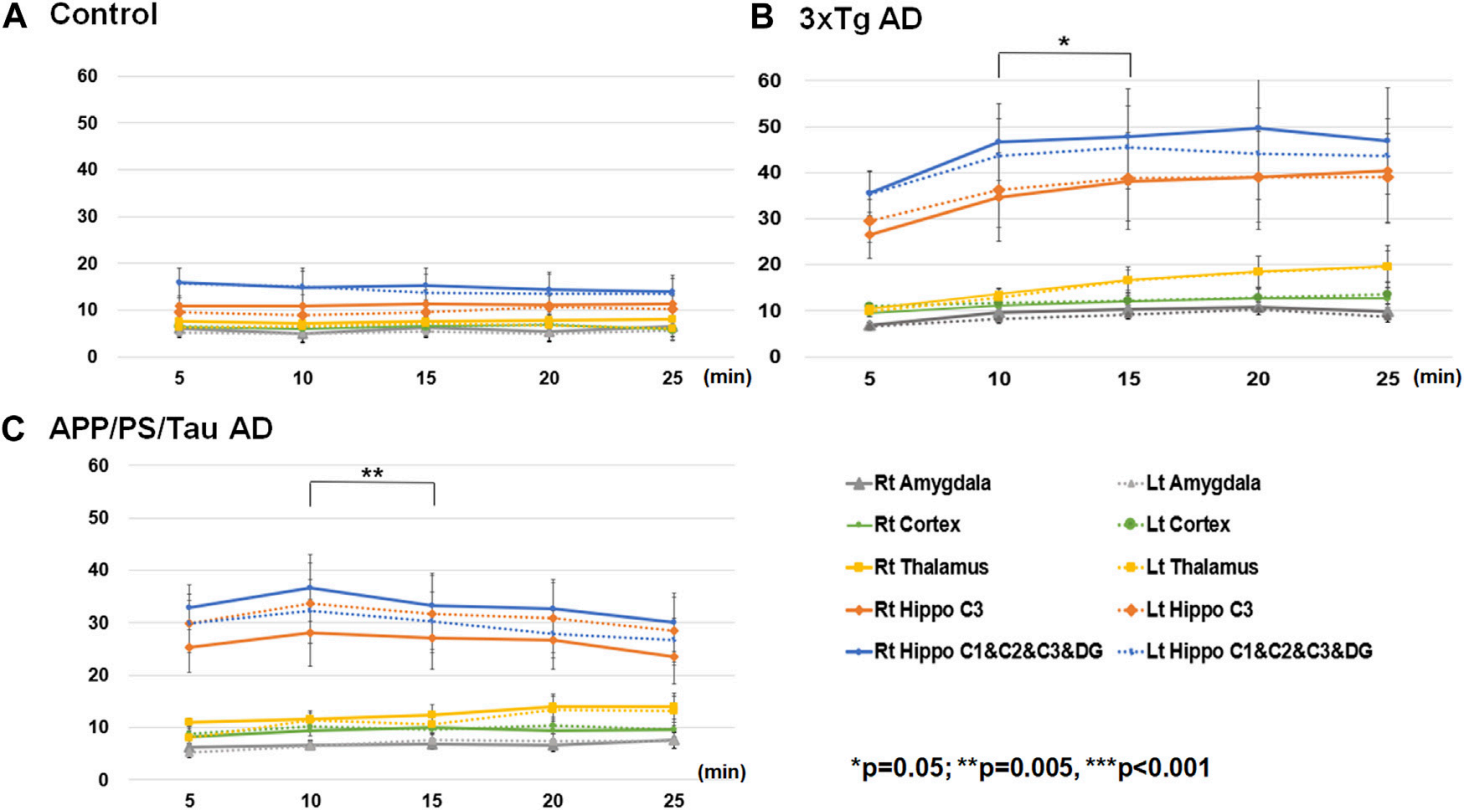
Result of region-of-interest (ROI)-based comparison of the signal enhancement between the scan-time points for Control **(A)**, 3xTg AD **(B)**, and App/PS/Tau AD **(C)** mouse groups. Data listed in Supplementary Table S5 as the median (25th −75th percentile) of the signal enhancement at the scanned times of 5, 10, 15, 20, and 25 min after injection of the contrast media for each mouse model.

Figure 6 shows the result of the ROI-based comparison of the signal enhancement between the scan-time points for each mouse model. Supplementary Table S5 also lists the median (25th −75th percentile) of the signal enhancement at the scanned times of 5, 10, 15, 20, and 25 min after injection of the contrast media for each mouse model and the result of the differences of the signal enhancement between the five different scan-time points for each mouse group.

For the control mouse group, there were no significant differences in signal enhancement between the scan-time points for any defined ROI areas. For the 3xTg mouse group, the signal enhancement was significantly different among the five scan-time points at the left thalamus (F = 4.366/*p* = 0.006). The signal enhancement was significantly different between 5 min and 25 min (*p* = 0.039), between 10 min and 15 min (*p* = 0.008), and between 10 min and 20 min (*p* = 0.020.) For the APP/PS/Tau mouse group, the signal enhancement was significantly different among the five scan-time points at the left amygdala F = 2.763/*p* = 0.040), left (F = 3.444/*p* = 0.016) and right (F = 4.508/*p* = 0.004) hippocampus C1&C2&C3&DG, and right hippocampus C3 (F = 4.474/*p* = 0.004). In the left amygdala, the signal enhancement was significantly different between 5 min and 15 min (*p* = 0.032). In the right hippocampus C1&C2&C3&DG, signal enhancement was significantly different between 10 min and 15 min (*p* = 0.003). In the right hippocampus C3, signal enhancement was significantly different between 10 min and 25 min (*p* = 0.024).

#### Comparison between low and high concentrations of the proposed contrast agent for only AD Tg mouse models

Table 3 shows the results of an ROI-based comparison of signal changes between low and high concentrations of the proposed contrast agent in each region-of-interest (ROI) brain area used only in the AD mouse models at each scanned time point. At 5 min scan-time point, the signal enhancement in the cortex was significantly higher with the low concentrations than with the high concentrations. At 10 min and 15 min scan-time points, the signal enhancement in the hippocampus C1&C2&C3&DG was significantly higher with the high concentrations than with the low concentrations. At the 20-minute scan-time point, the signal enhancement in the hippocampus C3 was significantly higher with the high concentrations than with the low concentrations. At the 25-min scan-time point, the signal enhancement was significantly higher with the high concentrations than with the low concentrations in the hippocampus C1&C2&C3&DG, hippocampus C3, thalamus, and amygdala. Data not listed in this table indicate that signals were not significantly different between the low (N = 11) and high (N = 9) concentrations of the proposed contrast agent: 1) in the hippocampus C1&C2C3&DG, hippocampus C3, thalamus, and amygdala at 5 min, 2) in the hippocampus C3, cortex, thalamus, and amygdala at both 10 and 15 min, 3) in the hippocampus C1&C2C3&DG, cortex, thalamus, and amygdala at 20 min, and 4) in the cortex at 25 min.

**TABLE 3:** Comparison of signal changes in each region-of-interest (ROI) brain area between low and high concentrations of the proposed contrast agent used only in the AD mouse models at each scanned time point.

Scan time (min)	ROI	Low concentration (N = 11)	High concentration (N = 9)	*p*-value
5	Cortex	10.892 (9.096–13.097)	7.843 (4.159–9.786)	0.014
10	HippoC1&C2C3&DG	21.539 (16.960–37.546)	43.487 (23.881–77.795)	0.021
15	HippoC1&C2C3&DG	17.562 (11.282–38.235)	45.505 (17.141–78.769)	0.044
20	HippoC3	16.673 (10.904–34.544)	43.543 (18.098–80.345)	0.021
25	HippoC1&C2C3&DG	18.170 (14.690–30.760)	40.904 (21.665–87.897)	0.017
	HippoC3	14.772 (10.324–31.518)	38.990 (16.433–79.960)	0.017
	Thalamus	10.847 (6.971–13.446)	22.804 (10.960–37.152)	0.025
	amygdala	5.990 (3.069–9.331)	12.487 (8.191–13.755)	0.014

Data list the median (95% confidence interval of median) value for the low and high concentrations of the proposed contrast agent used only in the AD, mouse models, which are both 3xTg and APP/PS/Tau.

Data not listed in this table indicate that signals were not significantly different between the low (N = 11) and high (N = 9) concentrations of the proposed contrast agent: 1) in the hippocampus C1&C2C3&DG, hippocampus C3, thalamus, and amygdala at 5 min, 2) in the hippocampus C3, cortex, thalamus, and amygdala at both 10 and 15 min, 3) in the hippocampus C1&C2C3&DG, cortex, thalamus, and amygdala at 20 min, and 4) in the cortex at 25 min.

### Immunofluorescence (IF) analysis

**FIGURE 7:**
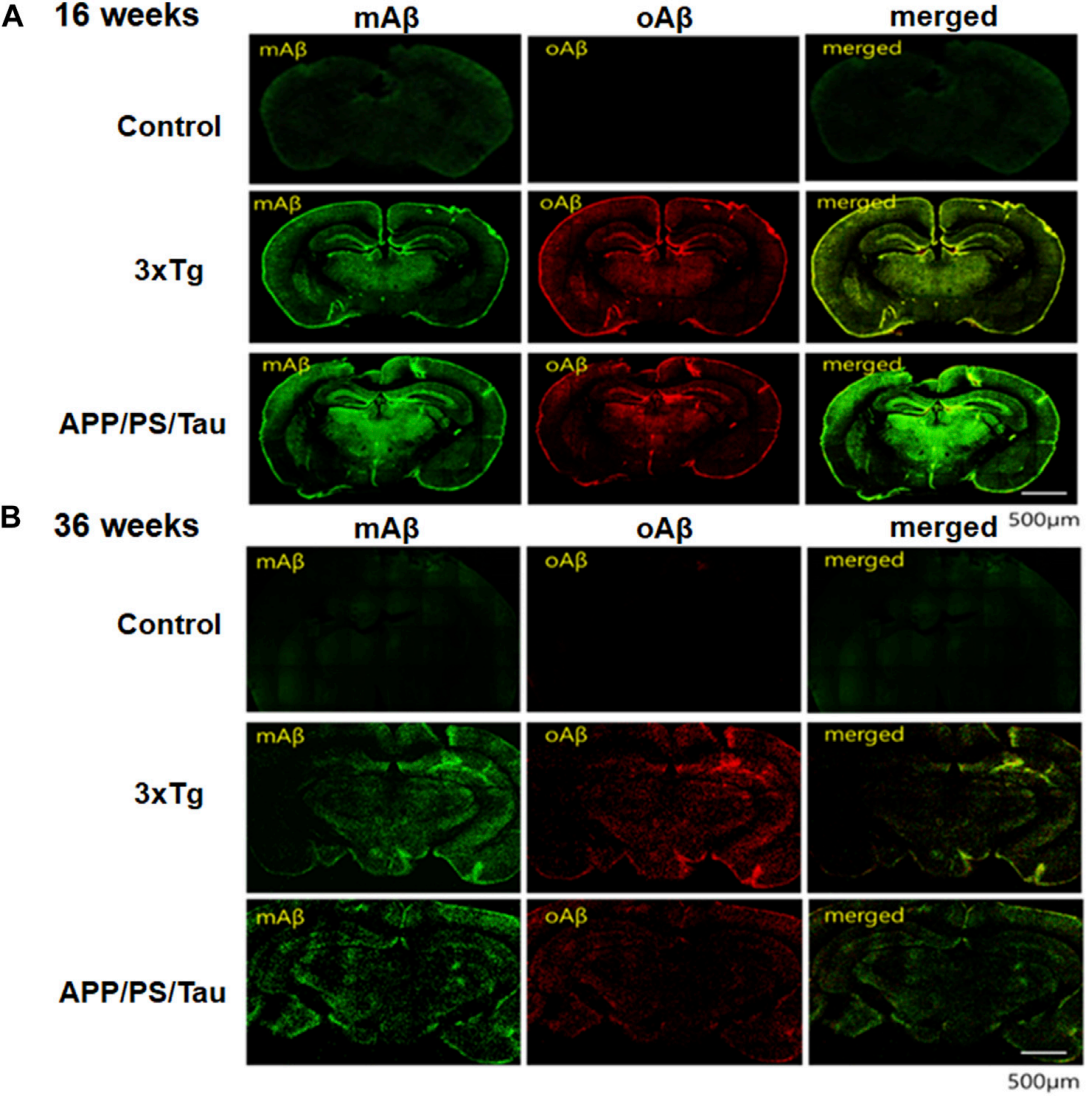
Results of immunofluorescence (IF) analysis of 16-week-old mice **(A)** and 36-week-old mice **(B)** of control, 3xTg AD, and APP/PS/Tau AD mouse models. The IF analysis showed the deposit pattern of monomer amyloid beta (mAβ) and oligomeric amyloid beta (oAβ) in the brain tissue of AD mouse models. Brain tissues were subjected to immunohistochemistry using antibodies to anti-mAβ (6E10, Biolegend, San Diego, CA, United States) and ob5 aptamer (J&Pharma, Korea) as indicated. The scale bar indicates 500 μm.

The IF analysis revealed the deposition pattern of mAβ and oAβ in the brain tissue of AD mouse models (Figure 7). The IF images of 16-week-old and 36-week-old 3xTg mice differed remarkably from those of non-Tg mice in each age group. Moreover, the amyloid deposition patterns in the brain of 16-week-old 3xTg AD or APP/PS/Tau AD mice were considerably different from those of 36-week-old 3xTg AD or APP/PS/Tau AD mice. In the non-Tg control group, there was almost no expression of mAβ and oAβ. However, in the 3xTg mice, red fluorescence due to oAβ was very strong, and green fluorescence due to mAβ was slightly strong, in the 16-week-old and 36-week-old mice, (Figure 7A).

On the other hand, to determine whether the signals were due to mAβ and oAβ and whether they were colocalized in the 36-week-old 3xTg mouse, we detected fluorescent anti-mAβAb-QD525 that showed strongly blue or green fluorescence under a confocal microscope and the fluorescent QD565-conjugated ob5 aptamer that showed slightly red fluorescence (Figure 7B). The results confirmed that mAβ and oAβ, which are a paralytic protein, were deposited at the same location in the 36-week-old 3xTg mouse, but oAβ deposits were more prominent using QD565-conjugated ob5 aptamer in the 16-week-old 3xTg mice. This finding is consistent with our previous observation that, in 3xTg mice, ApoE KO mutant accumulated more abundantly within brain tissues than control mice. These features indicate that the 3xTg AD mice are a suitable model for investigating oAβ deposits even before the accumulation of amyloid plaques at young ages.

We performed *in vitro* neuropathological fluorescent staining of mAβ and oAβ deposits in slices of brain tissue from 3xTg and APP/PS/Tau AD mice and control mice to evaluate the affinity of these monoclonal Aβ antibody (green) and ob5-cy5.5 (red) probes. As shown in Figure 7, the presence and distribution of mAβ were consistent with the results of staining adjacent slices using anti-mAβ (6E10) (Figures 7A,B), while no oAβ staining was found when using ob5 (Figure 7A) in C57B6BL mice, which further confirmed that ob5 had no affinity to mAβ. Furthermore, intense labeling of oAβ by ob5 was strongly observed in the brain slices of both AD mice (Figure 7A). More interestingly, Figure 7B showed slight staining of mAβ and oAβ in the cortex region of the normal and AD Tg mice (Figure 7B). *Ex vivo* intensity studies indicated that Figure 7A has high *ex vivo* oAβ deposits in mouse brain tissues of 16-week-old mice, and the fluorescence intensity decreased by an average of 55% in 36-week-old mice with two AD mice. There was a significant difference in the clearance profile after the intensity of 16-week-old mice between AD Tg and wild-type control mice, and the brain of AD mice exhibited synaptic alteration in parallel with intraneuronal deposits of Aβ oligomers without formation of amyloid plaques during early aged stage, which might be strongly attributed to the high affinity of ob5 to oAβ deposits in both AD mice.

#### Contrast enhancement patterns in 3xTg and APP/PSTau mouse models after injection of low- or high-contrast agents

**FIGURE 8:**
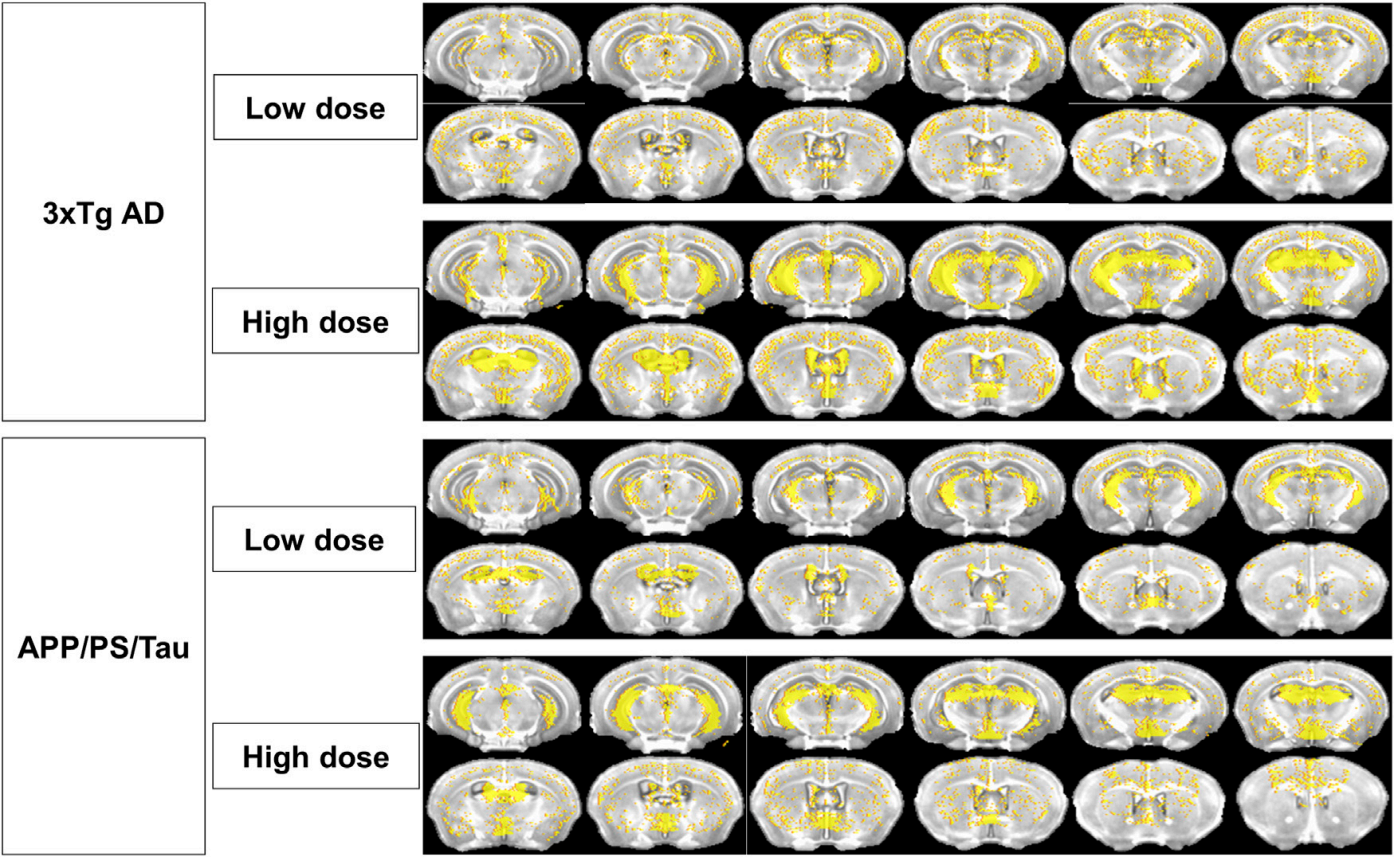
Distribution of signal enhancement in 3xTg AD and APP/PS/Tau mouse models after injection of low or high-contrast agents in 10 scan-time points. Signal enhancements in AD mouse models were concentrated in the hippocampus C1&C2&C3&DG, hippocampus C3, cortex, and thalamus in both low and high doses of the proposed contrast agent. Signal enhancements in high doses were more extensive than those in low doses.

Figure 8 showed the distribution of signal enhancement in 3xTg AD and APP/PS/Tau mouse models after injection of low or high dose of proposed contrast agent in 10 min. In both low and high doses, the signal enhancements in 3xTg AD and APP/PS/Tau mouse models were mainly concentrated on hippocampus C1&C2&C3&DG, hippocampus C3, cortex, and thalamus. There was less signal enhancement in the amygdala in two AD mouse models. In addition, signal enhancements in the AD mouse model in high doses were more extensive compared with signal enhancement in low doses.

## Discussion

The objective of this study was to evaluate the signal enhancement using our developed MRI contrast agent in AD mouse models. This contrast agent was developed to target oligomeric amyloid-beta for early diagnosis of AD using MRI (Kim et al., 2023). In this study, we evaluated the time-dependent contrast enhancement effect using the contrast agent in 3xTG AD and APP/PS1/Tau AD models at 16 or 36 weeks of age. The main findings of this study are as follows: (1) In both the 3xTG AD model and the APP/PS1/Tau AD model, the signal enhancement after injection of our developed contrast agent was observed from 16 weeks of age, confirming the potential for early diagnosis of AD. Specifically, AD models at 16 weeks showed higher contrast enhancement than those at 36 weeks. In both the 3xTG AD and APP/PS1/Tau AD models, the hippocampal area was the primary brain region where the contrast effect of the oligomeric amyloid-β targeted contrast agent was observed, with varying patterns of contrast effect depending on the model. (2) For 3xTg AD and APP/PS/Tau AD groups, the signal enhancement was significantly different among the five scan-time points in multiple ROI areas, indicating a delayed enhancement compared to a commercially available contrast agent, which is usually not specific in AD diagnosis.

### Signal enhancement at 16-week-old mice in the hippocampus area

We demonstrated that the T1-enhanced signal by the oligomeric amyloid-β targeted contrast agent was higher in 16-week-old AD models compared to the 36-week-old models. Our previous research proved that the oligomeric amyloid-β targeted contrast agent could visualize oligomeric amyloid-β in the 3xTG AD model (Kim et al., 2023). This suggests that the higher T1-enhanced signal in 16-week-old AD models indicates a greater presence of oligomeric amyloid-β in brain regions compared to the 36-week-old AD models. Recent studies have reported that the formation of oligomeric amyloid-β and Aβ plaques are pathologically interconnected in AD (Huang and Liu, 2020). Oligomeric amyloid-β is formed by the binding of a few amyloid-β proteins, which can later develop into amyloid plaques commonly found in the brains of AD patients (Lesne and Kotilinek, 2005; Sun et al., 2015; Mroczko et al., 2018). Numerous studies have developed MRI contrast agents and diagnostic radiopharmaceuticals for early AD diagnosis in animal brains (Bort et al., 2014; Patil et al., 2015; Zhang et al., 2015; Dudeffant et al., 2017; Dong et al., 2020; Luo et al., 2020), but to our knowledge, there were no MRI contrast agents or diagnostic radiopharmaceuticals available for detecting early oligomeric amyloid-β. Therefore, based on our findings, we suggest that the oligomeric amyloid-β targeted contrast agent can provide important information for the development of early diagnosis and treatment strategies for AD.

We confirmed a higher contrast effect in the hippocampus of the AD model compared to the control group through ROI-based analysis. Also, after the administration of the contrast agent, a pronounced statistical difference was observed only in the hippocampus of the AD model compared to the normal group, with no contrast effect differences in the cerebral cortex. This result differs from the typical amyloidosis process. Generally, in the early stages of AD, the accumulation of amyloid oligomers is observed mainly in specific areas of the cerebral cortex, such as the frontal and temporal lobes, and then extends to the limbic system like the hippocampus (Reilly et al., 2003; McGowan et al., 2006; Kimura and Ohno, 2009; Willuweit et al., 2009). Our findings, showing differences only in the hippocampus and not in typical amyloidosis, suggest a correlation with previous research indicating that cognitive impairment in long-term memory retention, which occurs in 16-week-old 3xTG AD, is related to amyloid-β accumulation, even though amyloid-β plaques are not present in the hippocampus and amygdala of 16-week-old 3xTG AD models (Billings et al., 2005). Furthermore, in 6-month-old 3xTG AD models, Aβ plaques first form in the cerebral cortex (Oddo et al., 2003). Therefore, in 36-week-old 3xTG AD models, a reduced amount of oligomeric amyloid-β may than amyloid plaques result in a lower contrast effect.

Although a high contrast effect was not observed in the cerebral cortex, the pronounced difference shown by our contrast agent in the hippocampus is important for evaluating AD models. The hippocampus plays a crucial role in memory formation and storage and shows high neural and metabolic activity, making it more vulnerable to oxidative stress and microenvironment changes (Venkateshappa et al., 2012; Watts et al., 2018; Rao et al., 2022). Therefore, the generation and accumulation of Aβ, especially in its oligomeric form, which is highly neurotoxic and can induce neuronal cell death and inflammatory responses, may be accelerated with the progression of AD (Sun et al., 2015). Consequently, observing AD pathology in the hippocampus at an early stage is extremely important, and among these, the evaluation of oligomers using contrast agents can be a useful indicator.

### Time-dependent signal enhancement pattern after injecting our contrast agent

After the administration of the contrast agent, time-dependent variations in the contrast effect in the hippocampus differed between the 3xTG AD and APP/PS/Tau AD animal models. In the 3xTG AD model, the signal increase after contrast agent administration was sustained up to 25 min. However, in the APP/PS/Tau AD model, the peak contrast effect was observed at around 10 min, followed by a continuous decrease. Although no statistical difference was found in ROI-based analysis between 3xTG AD and APP/PS/Tau AD, the variation in the contrast effect suggests it may reflect the characteristics of the animal models. In the early pathological stages of AD, oligomeric amyloid-β tends to accumulate more around cells rather than inside them, showing the formation of aggregates in the extracellular space (Mroczko et al., 2018; Huang and Liu, 2020). Our previous research indicated that the late enhancement of the contrast effect was due to oligomeric amyloid-β circulating around amyloid plaques, and the rapid increase and decrease in the contrast effect were attributed to oligomeric amyloid-β in the brain’s extracellular space (Kim et al., 2023). Therefore, the initial rapid increase in contrast effect in both 3xTG AD and APP/PS/Tau AD could be due to an increase in oligomeric amyloid-β in the extracellular space in the early stages of AD. Additionally, the ongoing increase in the contrast effect in 3xTG AD over time and the decrease in APP/PS/Tau AD suggest a difference in the amount of oligomeric amyloid-β circulating around the amyloid plaques, indicating a more dominant formation of amyloid plaques in 3xTG AD compared to APP/PS/Tau AD Hence, our contrast agent proposes the flexibility in evaluating differences due to amyloid oligomers, suggesting its translational potential across model species.

### Possible mechanism of interaction between oAβ and a DNA aptamer

The interaction between oligomeric amyloid-beta (Aβ) and a DNA aptamer involves a specific binding mechanism where the aptamer selectively recognizes and binds to the Aβ oligomer. The interaction of the process between them may be explained as the following: First, DNA aptamers are single-stranded DNA molecules that can fold into unique three-dimensional structures (Jeddi and Saiz, 2017). These structures are designed to have high affinity and specificity for their target molecules, in this case, the Aβ oligomer (Zhou and Rossi, 2017; Sharma et al., 2022). Second, when the DNA aptamer encounters an Aβ oligomer, it undergoes a conformational change that allows it to fit snugly around the oligomer. This is often due to the formation of complementary shapes and charge interactions between the aptamer and the specific epitopes on the Aβ oligomer (Guo et al., 2023). Upon binding, the aptamer may wrap around the Aβ oligomer or form a pocket that encloses it (Deng et al., 2020). This conformational change is critical as it brings the aptamer’s nucleotides into proximity with the Aβ oligomer, allowing for hydrogen bonding and electrostatic interactions. Third, the binding is stabilized by various non-covalent interactions, such as hydrogen bonds, van der Waals forces, and electrostatic attractions (Yu et al., 2023). These interactions ensure that the aptamer remains tightly bound to the Aβ oligomer. The specificity of the aptamer for the Aβ oligomer is due to the precise arrangement of its nucleotides, which are selected through an iterative process known as SELEX (Systematic Evolution of Ligands by EXponential enrichment) (Li and Lee, 2019). This process yields aptamers that are highly specific to their target molecules. Finally, the binding of the aptamer to the Aβ oligomer can inhibit the oligomer’s pathological interactions with cellular components, potentially preventing the toxic effects associated with Alzheimer’s disease (Zheng et al., 2022). For instance, an electrochemical aptasensor developed for Aβ oligomers utilizes a double-stranded DNA as a “conductive spring” (Deng et al., 2020; Ren et al., 2020). Upon the binding of the aptamer to the Aβ oligomer, the conformation of the aptamer changes, leading to a measurable change in the electrical signal, which can be correlated to the concentration of Aβ oligomers. The specificity and sensitivity of DNA aptamers make them suitable for detecting Aβ oligomers, which are considered key biomarkers and therapeutic targets in the pathology of Alzheimer’s disease (Ren et al., 2020; Zheng et al., 2022).

### Study limitations

This study had several limitations. First, we did not compare the developed contrast agent with a commercially available MRI contrast agent, which is currently not used for AD diagnosis. This limits the generalizability of our findings and the potential clinical applications of our contrast agent. Second, we only used two types of AD mouse models, 3xTG and APP/PS/Tau, while there are several other types of animal models for AD such as 5xFAD. Therefore, the new developed contrast agent should be evaluated with more AD model mice to assess its validity and reliability across different models. Third, we did not include amyloid-beta PET, which is usually targeted to amyloid plaques rather than oligomeric amyloid-beta. Therefore, a direct comparison between the two imaging modalities might not be adequate to show the distribution of oligomeric amyloid-beta in the AD model mice brain. A multimodal imaging approach might be more informative and comprehensive for detecting AD pathology. Finally, the number of mice in each model was relatively small, which might reduce the statistical power and the sensitivity of our analyses. Therefore, we recommend a more extensive study with more mice in each model and more AD models to confirm and extend our results.

## Conclusion

The findings of this study suggested that the expression of the contrast agent in different AD models demonstrated that the agent can reflect the degree of oligomeric amyloid-β expression as a contrast enhancement regardless of the AD model, demonstrating its translational flexibility across different species. In addition, this study demonstrated that the signal enhancement was higher at 16 weeks of age than at 36 weeks of age, thus proving its potential for early diagnosis of AD. Furthermore, our study showed that the signal enhancement peaked around 15–20 min after injection of the contrast agent, indicating a delayed enhancement compared to a commercially available contrast agent, which is usually not specific for diagnosis of Alzheimer’s disease. Therefore, our novel contrast agent targeting oAβ has the potential ability to diagnose early AD and monitor the progression of AD because our contrast agent targeting oligomeric amyloid-β can visualize oligomeric amyloid-β occurring early in AD.

## Data Availability

The raw data supporting the conclusion of this article will be made available by the authors, without undue reservation.

## Abbreviations

AD, Alzheimer’s disease; Aβ, amyloid beta; BBB, blood brain barrier; BW, bandwidth; CPMG, Carr-Purcell-Meiboom-Gill; cy5, cyanine5; DOTA, dodecane tetraacetic acid; FOV, field of view; IHC, immunohistochemical; (KO), , Konckout; Gd, Gadolinium; Aβ (mAβ), monomer; MRI, magnetic resonance imaging; NSA, number of averages; oAβ, oligomeric Aβ; PET, positron emission tomography; PrP, prion proteins; ROI, region-of-interest; SELEX, Selective Evolution of Ligands by Exponential enrichment; SNR, signal-to-noise ratio; TE, echo time; Tg, Transgenic; TR, repetition time; [^11^C] PiB, [^11^C] Pittsburgh Compound-B.
