# Supplementary material for: Oligomeric amyloid-β targeted contrast agent for MRI evaluation of Alzheimer’s disease mouse models
Source: Front Pharmacol. 2024 Jun 4;15:1392729. doi: 10.3389/fphar.2024.1392729 (PMC11184063; doi:10.3389/fphar.2024.1392729)
Supplement: Supplementary file 1 [file DataSheet1.docx]

**Supplementary Tables**

**Supplementary Table S1. Results of the group differences of the signal enhancement between the three different mouse models for each scan-time point.**

| **ROI** | **Control (1)** | **3xTg (2)** | **APP/PS/Tau (3)** | ***p-value** |
| --- | --- | --- | --- | --- |
| **Post 5min** | | | | |
| Amygdala Lt | 4.69 (3.30-8.44) | 6.03 (5.29-6.85) | 4.84 (2.31-7.07) | F= 0.720, P= 0.700 |
| Amygdala Rt | 6.30 (4.34-8.41) | 6.46 (5.71-8.74) | 5.65 (3.46-8.73) | F= 0.322, P= 0.851 |
| Cortex Lt | 6.89 (3.70-10.56) | 10.39 (8.23-13.07) | 8.90(7.06-12.69) | F= 3.903, P= 0.142 |
| Cortex Rt | 7.39 (3.24-10.17) | 9.89 (7.96-11.11) | 9.41 (6.51-12.29) | F= 1.953, P= 0.377 |
| Hippo_C1&C2&C3&DG Lt | 16.05 (9.08-26.65) | 33.04 (25.49-47.55) | 21.77 (18.79-42.60) | F= 7.677, P= 0.022  (p= 0.010, 1,2)  (p= 0.041, 1,3) |
| Hippo_C1&C2&C3&DG Rt | 15.92 (10.15-24.26) | 37.47 (23.84-51.00) | 28.47 (19.12-47.02) | F= 8.763, P= 0.012  (p= 0.011, 1,2)  (p= 0.011, 1,3) |
| Hippo_C3 Lt | 10.33 (7.98-14.67) | 27.48 (16.93-40.73) | 20.99 (14.11-43.03) | F =13.774, P= 0.001  (p< 0.001, 1,2)  (p= 0.003, 1,3) |
| Hippo_C3 Rt | 10.91 (6.90-16.70) | 21.93 (15.75-34.12) | 18.44 (15.80-38.90) | F= 8.132, P= 0.017  (p= 0.006, 1,2)  (p= 0.041, 1,3) |
| Thalamus Lt | 6.98 (3.12-10.77) | 10.05 (7.67-12.07) | 10.00 (4.62-11.55) | F= 2.422, P= 0.297 |
| Thalamus Rt | 8.24 (5.33-11.21) | 11.22 (8.33-12.00) | 12.38 (9.24-12.98) | F= 3.886, P= 0.143 |
| **Post 10min** | | | | |
| Amygdala Lt | 6.25 (3.04-9.64) | 7.99 (7.31-9.88) | 6.33 (4.53-8.54) | F= 2.164, P= 0.339 |
| Amygdala Rt | 7.64 (1.18-8.52) | 9.66 (7.83-11.26) | 6.89 (4.13-9.18) | F= 5.438, P= 0.066 |
| Cortex Lt | 7.50 (5.12-11.27) | 12.55 (9.76-14.45) | 11.03 (9.95-12.39) | F= 5.391, P= 0.068 |
| Cortex Rt | 8.96 (3.34-9.96) | 11.50 (8.75-13.64) | 9.54 (7.25-12.14) | F= 4.302, P= 0.116 |
| Hippo_C1&C2&C3&DG Lt | 17.07 (9.98-21.69) | 36.54 (25.31-68.54) | 29.03 (17.39-60.91) | F= 10.648, P= 0.005  (p= 0.011, 1,2) |
| Hippo_C1&C2&C3&DG Rt | 17.07 (9.98-21.69) | 36.54 (25.31-68.54) | 29.03 (17.39-60.91) | F= 10.648, P= 0.005  (p= 0.002, 1,2)  (p= 0.035, 1,3) |
| Hippo_C3 Lt | 11.26 (8.19-17.28) | 21.58 (15.36-49.03) | 17.96 (13.08-41.42) | F= 6.098, P= 0.047  (p= 0.003, 1,2)  (p= 0.005, 1,3) |
| Hippo_C3 Rt | 11.26 (8.19-17.28) | 21.58 (15.36-49.03) | 17.96 (13.08-41.42) | F= 6.098, P= 0.047  (p= 0.018, 1,2) |
| Thalamus Lt | 8.14 (5.77-11.09) | 11.55 (8.89-16.82) | 11.30 (9.49-14.02) | F= 5.552, P= 0.062 |
| Thalamus Rt | 7.84 (5.47-10.42) | 11.69 (9.93-16.22) | 12.39 (11.12-13.54) | F= 4.481, P= 0.106 |
| **Post 15min** | | | | |
| Amygdala Lt | 6.77 (2.41-9.69) | 7.76 (7.12-10.97) | 8.23 (4.74-10.59) | F= 2.113, P= 0.348 |
| Amygdala Rt | 8.01 (4.66-10.09) | 11.92 (6.91-12.99) | 7.68 (4.36-9.54) | F= 3.372, P= 0.185 |
| Cortex Lt | 7.57 (4.89-10.37) | 11.55 (8.55-16.25) | 9.68 (7.98-12.88) | F= 4.864, P= 0.088 |
| Cortex Rt | 9.19 (5.05-10.18) | 12.11 (9.09-15.47) | 10.48 (7.93-12.34) | F= 5.391, P= 0.068 |
| Hippo_C1&C2&C3&DG Lt | 16.31 (5.70-19.85) | 32.02 (24.72-71.91) | 19.99 (16.14-42.31) | F= 10.227, P= 0.006  (p= 0.003, 1,2) |
| Hippo_C1&C2&C3&DG Rt | 18.22 (9.34-22.30) | 36.97 (21.51-74.23) | 24.66 (13.61-54.48) | F= 6.287, P= 0.043  (p= 0.018, 1,2) |
| Hippo_C3 Lt | 18.22 (9.34-22.30) | 36.97 (21.51-74.23) | 24.66 (13.61-54.48) | F= 6.257, P= 0.043  (p= 0.007, 1,2)  (p= 0.024, 1,3) |
| Hippo_C3 Rt | 11.42 (9.13-16.55) | 21.99 (12.07-55.87) | 16.83 (14.18-40.64) | F= 5.680, P= 0.058 |
| Thalamus Lt | 9.87 (5.88-11.17) | 13.88 (10.71-21.76) | 9.31 (6.75-14.16) | F= 6.832, P= 0.033  (p=0.011, 1,2) |
| Thalamus Rt | 7.46 (6.19-10.81) | 13.57 (10.27-23.98) | 13.05 (9.84-13.65) | F= 5.191, P= 0.075 |
| **Post 20min** | | | | |
| Amygdala Lt | 5.24 (2.82-9.35) | 9.88 (7.00-12.62) | 6.92 (4.10-9.28) | F= 5.294, P= 0.071 |
| Amygdala Rt | 7.67 (1.03-9.35) | 12.18 (7.64-13.55) | 6.79 (3.54-7.48) | F= 5.766, P= 0.056 |
| Cortex Lt | 7.55 (4.06-11.57) | 12.00 (8.43-19.13) | 10.12 (5.76-13.86) | F= 3.925, P= 0.141 |
| Cortex Rt | 8.59 (4.65-11.15) | 11.62 (8.58-17.86) | 9.87 (5.55-11.88) | F= 3.006, P= 0.222 |
| Hippo_C1&C2&C3&DG Lt | 16.19 (4.16-21.00) | 31.31 (19.83-74.15) | 25.62 (16.25-38.06) | F= 6.708, P= 0.035  (p= 0.014, 1,2) |
| Hippo_C1&C2&C3&DG Rt | 17.26 (9.49-20.71) | 36.58 (21.72-77.71) | 25.76 (17.48-45.05) | F= 8.362, P= 0.015  (p= 0.009, 1,2)  (p= 0.029, 1,3) |
| Hippo_C3 Lt | 11.05 (7.33-15.19) | 28.48 (14.27-57.00) | 29.96 (14.06-41.28) | F= 7.855, P= 0.020  (p= 0.018, 1,2)  (p= 0.018, 1,3) |
| Hippo_C3 Rt | 11.00 (7.83-18.18) | 21.37 (12.29-62.15) | 18.16 (13.88-37.87) | F= 5.555, P= 0.062 |
| Thalamus Lt | 7.28 (4.71-11.72) | 16.39 (9.04-27.43) | 11.51(8.24-18.63) | F= 5.534, P= 0.063 |
| Thalamus Rt | 9.49 (6.15-11.16) | 11.66 (9.67-26.89) | 12.83 (10.42-17.38) | F= 4.725, P= 0.094 |
| **Post 25min** | | | | |
| Amygdala Lt | 7.37 (3.82-10.12) | 9.77 (5.18-11.87) | 9.16 (4.50-10.21) | F= 1.281, P= 0.527 |
| Amygdala Rt | 8.60 (4.02-10.65) | 12.93 (4.23-14.44) | 8.86 (4.19-10.21) | F= 1.029, P= 0.598 |
| Cortex Lt | 7.18 (5.50-10.02) | 14.45 (7.38-18.35) | 8.66 (6.36-13.76) | F= 5.161, P= 0.076 |
| Cortex Rt | 8.76 (2.50-11.00) | 14.29 (7.05-19.43) | 9.89 (7.82-13.18) | F= 3.102, P= 0.212 |
| Hippo_C1&C2&C3&DG Lt | 16.29 (6.16-18.43) | 26.74 (18.67-76.26) | 21.15 (16.24-33.61) | F= 6.943, P= 0.031  (p= 0.014, 1,2) |
| Hippo_C1&C2&C3&DG Rt | 17.23 (9.90-20.19) | 30.16 (16.98-77.01) | 23.59 (16.52-42.51) | F= 4.989, P= 0.083 |
| Hippo_C3 Lt | 9.96 (9.17-14.40) | 23.95 (13.52-57.29) | 16.54 (14.19-37.90) | F= 7.554, P= 0.023  (p= 0.022, 1,2)  (p= 0.017, 1,3) |
| Hippo_C3 Rt | 11.25 (9.24-16.30) | 21.21 (13.92-65.48) | 16.32 (13.71-37.68) | F= 5.279, P= 0.071 |
| Thalamus Lt | 8.76 (4.94-9.86) | 14.91 (10.07-29.76) | 11.00 (7.49-19.21) | F= 7.533, P= 0.023  (p=0.009, 1,2) |
| Thalamus Rt | 9.94 (7.37-12.60) | 12.77 (8.75-30.98) | 12.79 (9.31-17.49) | F= 3.423, P= 0.181 |

Data list median (25th -75th percentile) of the signal enhancement calculated by (Smin-Spre)*100%/ Spre, where Smin is the signal at the scanned times of 5, 10, 15, 20, and 25min and Spre is the signal acquired before injection of the contrast media.

*P-value by Kruskal-Wallis test with the post-hoc analysis using Mann-Whitney test after Kruskal-Wallis test

Abbreviations: DG, Dentate gyrus; Lt, left; Rt, right; ROI, region of interest

**Supplementary Table S2. Results of the comparison of the signal enhancement for each time point between 16 week-aged and 36 week-aged mice using both Tg mouse models**

| **ROI** | **16 weeks** | **36 weeks** | **p-value** |
| --- | --- | --- | --- |
| **Post 5min** | | | |
| Amygdala Lt | 5.00 (3.02-6.41) | 6.41 (5.33-7.12) | p= 0.257 |
| Amygdala Rt | 5.85 (4.30-6.46) | 8.51 (5.30-9.63) | p= 0.096 |
| Cortex Lt | 10.12 (8.79-13.52) | 8.71 (7.95-11.54) | p= 0.257 |
| Cortex Rt | 9.60 (7.35-12.32) | 10.14 (6.01-11.07) | p= 0.545 |
| Hippo_C1&C2&C3&DG Lt | 38.78 (25.67-48.52) | 23.36 (18.53-33.04) | p= 0.131 |
| Hippo_C1&C2&C3&DG Rt | 46.62 (32.97-51.61) | 25.48 (16.33-28.47) | P= 0.028 |
| Hippo_C3 Lt | 38.60 (27.48-45.01) | 16.89 (14.66-20.99) | P= 0.028 |
| Hippo_C3 Rt | 32.33 (17.45-47.70) | 17.89 (10.64-21.93) | P= 0.112 |
| Thalamus Lt | 10.72 (8.11-12.31) | 9.38 (3.87-10.09) | P= 0.227 |
| Thalamus Rt | 11.45 (9.11-12.38) | 11.99 (8.62-13.03) | P= 0.650 |
| **Post 10min** | | | |
| Amygdala Lt | 6.89 (5.76-9.93) | 7.24 (6.69-9.06) | P= 0.940 |
| Amygdala Rt | 7.11 (4.89-10.88) | 7.96 (7.55-9.66) | P= 0.545 |
| Cortex Lt | 11.95 (10.03-14.32) | 11.51 (9.38-12.53) | p= 0.257 |
| Cortex Rt | 11.03 (7.89-13.51) | 10.25 (7.71-12.34) | p= 0.597 |
| Hippo_C1&C2&C3&DG Lt | 42.31 (25.50-59.92) | 22.01 (16.26-30.83) | p= 0.041 |
| Hippo_C1&C2&C3&DG Rt | 57.39 (34.80-65.73) | 24.60 (17.33-30.07) | p= 0.041 |
| Hippo_C3 Lt | 45.02 (38.37-59.57) | 15.94 (13.53-24.98) | p= 0.041 |
| Hippo_C3 Rt | 39.18 (14.30-60.70) | 17.07 (15.69-20.99) | p= 0.174 |
| Thalamus Lt | 14.55 (9.29-18.71) | 10.88 (9.02-11.46) | p= 0.082 |
| Thalamus Rt | 13.43 (11.72-16.07) | 11.59 (10.04-13.51) | p= 0.174 |
| **Post 15min** | | | |
| Amygdala Lt | 8.16 (7.28-10.71) | 8.38 (6.28-10.78) | p= 0.880 |
| Amygdala Rt | 8.52 (6.99-11.92) | 7.30 (6.54-11.47) | p= 0.650 |
| Cortex Lt | 13.26 (8.77-15.75) | 9.23 (7.91-11.55) | p= 0.151 |
| Cortex Rt | 12.00 (8.99-16.03) | 10.37 (9.12-12.11) | p= 0.326 |
| Hippo_C1&C2&C3&DG Lt | 41.94 (30.84-69.45) | 20.34 (15.80-26.29) | p= 0.019 |
| Hippo_C1&C2&C3&DG Rt | 56.36 (31.05-67.62) | 22.43 (13.10-24.66) | p= 0.049 |
| Hippo_C3 Lt | 46.42 (39.60-52.09) | 18.03 (12.40-22.05) | p= 0.070 |
| Hippo_C3 Rt | 40.28 (12.13-53.49) | 15.96 (13.92-20.83) | p= 0.227 |
| Thalamus Lt | 15.94 (8.31-20.30) | 10.49 (8.71-13.79) | p= 0.227 |
| Thalamus Rt | 14.96 (11.83-22.46) | 11.19 (9.44-13.38) | p= 0.131 |
| **Post 20min** | | | |
| Amygdala Lt | 8.33 (5.82-12.07) | 8.15 (6.92-11.60) | p= 0.650 |
| Amygdala Rt | 7.31 (4.87-13.48) | 7.49 (6.79-12.70) | p= 0.880 |
| Cortex Lt | 13.08 (7.51-18.04) | 10.85 (7.75-13.72) | p= 0.545 |
| Cortex Rt | 9.41 (7.19-17.67) | 10.04 (8.68-12.32) | p= 0.940 |
| Hippo_C1&C2&C3&DG Lt | 39.86 (27.87-67.00) | 18.68 (14.01-25.62) | p= 0.019 |
| Hippo_C1&C2&C3&DG Rt | 53.45 (29.35-65.43) | 23.37 (18.31-25.76) | p= 0.082 |
| Hippo_C3 Lt | 46.24 (34.64-51.60) | 16.43 (13.55-28.48) | p= 0.070 |
| Hippo_C3 Rt | 38.31 (12.49-53.21) | 17.29 (13.84-21.37) | p= 0.257 |
| Thalamus Lt | 17.24 (7.92-25.29) | 11.21 (9.21-16.39) | p= 0.406 |
| Thalamus Rt | 16.07 (11.87-24.14) | 11.47 (9.94-13.07) | p= 0.257 |
| **Post 25min** | | | |
| Amygdala Lt | 9.33 (5.90-11.92) | 8.44 (5.06-10.51) | p= 0.545 |
| Amygdala Rt | 10.10 (3.80-14.26) | 7.81 (4.54-13.96) | p= 0.650 |
| Cortex Lt | 13.35 (7.36-17.87) | 10.25 (6.75-13.95) | p= 0.450 |
| Cortex Rt | 12.88 (5.73-19.23) | 9.43 (7.71-13.53) | p= 0.406 |
| Hippo_C1&C2&C3&DG Lt | 36.23 (20.84-61.51) | 19.41 (14.30-26.08) | p= 0.034 |
| Hippo_C1&C2&C3&DG Rt | 52.78 (23.59-65.27) | 19.36 (15.79-24.73) | p= 0.070 |
| Hippo_C3 Lt | 44.18 (30.41-52.85) | 15.31 (14.09-20.90) | p= 0.082 |
| Hippo_C3 Rt | 40.68 (10.38-57.19) | 15.22 (13.80-17.88) | p= 0.257 |
| Thalamus Lt | 20.71 (8.21-28.38) | 10.90 (7.87-14.91) | p= 0.326 |
| Thalamus Rt | 19.12 (12.79-27.56) | 10.96 (8.94-12.77) | p= 0.112 |

Data list median (25th -75th percentile) of the signal enhancement calculated by (Smin-Spre)*100%/ Spre, where Smin is the signal at the scanned times of 5, 10, 15, 20, and 25min and Spre is the signal acquired before injection of the contrast media.

*P-value by Mann-Whitney test

**Supplementary Table S3. Results of the comparison of the signal enhancement for each time point between 16-weeks-aged control and 16 weeks-aged both Tg mouse models**

| **ROI** | **16 weeks Control** | **16 weeks All Tg** | **p-value** |
| --- | --- | --- | --- |
| **Post 5min** | | | |
| Amygdala Lt | 5.00 (3.02-6.41) | 8.42 (5.42-8.71) | p= 0.129 |
| Amygdala Rt | 5.85 (4.30-6.46) | 7.75 (5.46-8.40) | p= 0.278 |
| Cortex Lt | 8.10 (3.70-10.56) | 10.12 (8.79-13.52) | p= 0.083 |
| Cortex Rt | 9.08 (3.24-10.17) | 9.60 (7.35-12.32) | p= 0.588 |
| Hippo_C1&C2&C3&DG Lt | 24.33 (13.64-27.17) | 38.78 (25.67-48.52) | p= 0.083 |
| Hippo_C1&C2&C3&DG Rt | 23.02 (10.15-26.36) | 46.62 (32.97-51.61) | p= 0.013 |
| Hippo_C3 Lt | 11.68 (9.62-14.67) | 38.60 (27.48-45.01) | p= 0.002 |
| Hippo_C3 Rt | 13.70 (9.16-16.70) | 31.33 (17.45-47.70) | p= 0.017 |
| Thalamus Lt | 7.81 (3.34-10.77) | 10.72 (8.11-12.31) | p= 0.193 |
| Thalamus Rt | 9.81 (5.33-11.45) | 11.45 (9.11-12.38) | p= 0.329 |
| **Post 10min** | | | |
| Amygdala Lt | 9.07 (3.74-9.70) | 6.89 (5.76-9.92) | p= 1.000 |
| Amygdala Rt | 7.11 (4.89-10.88) | 8.11 (1.18-8.52) | p= 0.828 |
| Cortex Lt | 9.12 (5.12-11.27) | 11.95 (10.03-14.32) | p= 0.129 |
| Cortex Rt | 9.43 (3.34-10.60) | 11.03 (7.89-13.51) | p= 0.278 |
| Hippo_C1&C2&C3&DG Lt | 21.18 (12.20-31.14) | 42.31 (25.50-59.92) | p= 0.023 |
| Hippo_C1&C2&C3&DG Rt | 21.55 (12.40-22.95) | 57.39 (34.80-65.72) | p= 0.017 |
| Hippo_C3 Lt | 11.61 (9.45-13.04) | 45.02 (38.37-59.57) | p= 0.009 |
| Hippo_C3 Rt | 14.33 (8.97-18.44) | 39.18 (14.30-60.70) | p= 0.051 |
| Thalamus Lt | 9.31 (5.77-11.24) | 14.55 (9.29-18.71) | p= 0.039 |
| Thalamus Rt | 9.29 (6.29-13.89) | 13.43 (11.72-16.07) | p= 0.193 |
| **Post 15min** | | | |
| Amygdala Lt | 8.04 (3.21-10.09) | 8.16 (7.28-10.71) | p= 0.588 |
| Amygdala Rt | 9.57 (4.66-11.53) | 8.52 (6.99-11.92) | p= 0.914 |
| Cortex Lt | 7.76 (5.33-11.99) | 13.26 (8.77-15.75) | p= 0.129 |
| Cortex Rt | 9.19 (5.05-11.03) | 12.00 (8.99-16.03) | p= 0.104 |
| Hippo_C1&C2&C3&DG Lt | 31.40 (13.22-27.64) | 41.94 (30.84-69.45) | p= 0.009 |
| Hippo_C1&C2&C3&DG Rt | 19.96 (1305-24.60) | 56.36 (31.05-67.62) | p= 0.030 |
| Hippo_C3 Lt | 12.08 (8.57-13.58) | 46.42 (39.60-52.09) | p= 0.017 |
| Hippo_C3 Rt | 13.44 (9.13-20.38) | 40.28 (12.13-53.49) | p= 0.065 |
| Thalamus Lt | 9.87 (5.88-11.92) | 15.94 (8.31-20.29) | p= 0.083 |
| Thalamus Rt | 9.15 (7.19-13.50) | 14.96 (11.83-22.46) | p= 0.104 |
| **Post 20min** | | | |
| Amygdala Lt | 8.71 (3.56-10.42) | 8.33 (5.82-12.07) | p= 0.828 |
| Amygdala Rt | 9.20 (1.03-9.46) | 7.31 (4.87-13.48) | p= 0.914 |
| Cortex Lt | 9.10 (6.66-11.59) | 13.08 (7.51-18.04) | p= 0.233 |
| Cortex Rt | 8.59 (5.14-11.15) | 9.41 (7.19-17.67) | p= 0.515 |
| Hippo_C1&C2&C3&DG Lt | 17.59 (9.91-28.68) | 39.86 (27.87-67.00) | p= 0.017 |
| Hippo_)C1&C2&C3&DG Rt | 19.10 (9.98-23.60) | 53.45 (29.35-65.43) | p= 0.030 |
| Hippo C3 Lt | 12.52 (7.33-18.23) | 46.24 (34.64-51.60) | p= 0.017 |
| Hippo C3 Rt | 12.83 (8.22-19.28) | 38.31 (12.49-53.21) | p= 0.065 |
| Thalamus Lt | 8.68 (5.08-11.72) | 17.24 (7.92-25.29) | p= 0.104 |
| Thalamus Rt | 10.08 (7.52-14.45) | 16.07 (11.87-24.14) | p= 0.129 |
| **Post 25min** | | | |
| Amygdala Lt | 9.90 (4.37-10.74) | 9.33 (5.90-11.92) | p= 1.000 |
| Amygdala Rt | 10.38 (4.02-12.40) | 10.10 (3.80-14.26) | p= 0.828 |
| Cortex Lt | 8.36 (5.50-10.02) | 13.35 (7.36-17.87) | p= 0.193 |
| Cortex Rt | 8.76 (2.50-9.31) | 12.88 (5.73-19.23) | p= 0.159 |
| Hippo_C1&C2&C3&DG Lt | 17.31 (11.97-28.11) | 36.23 (20.84-61.51) | p= 0.013 |
| Hippo_C1&C2&C3&DG Rt | 18.22 (10.48-25.33) | 52.78 (23.59-65.27) | p= 0.039 |
| Hippo_C3 Lt | 12.39 (9.25-18.60) | 44.18 (30.41-52.85) | p= 0.017 |
| Hippo_C3 Rt | 12.76 (9.24-19.53) | 40.68 (10.38-57.19) | p= 0.065 |
| Thalamus Lt | 9.63 (6.61-10.61) | 20.71 (8.21-28.38) | p= 0.083 |
| Thalamus Rt | 11.20 (7.73-13.63) | 19.12 (12.79-27.56) | p= 0.065 |

Data list median (25th -75th percentile) of the signal enhancement calculated by (Smin-Spre)*100%/ Spre, where Smin is the signal at the scanned times of 5, 10, 15, 20, and 25min and Spre is the signal acquired before injection of the contrast media.

*P-value by Mann-Whitney test

**Supplementary Table S4. Results of the comparison of the signal enhancement for each time point between 36-weeks-aged control and 36 weeks-aged both Tg mouse models**

| **ROI** | **Control** | **All Tg-36 weeks** | **p-value** |
| --- | --- | --- | --- |
| **Post 5min** | | | |
| Amygdala Lt | 3.50 (1.87-3.83) | 6.41 (5.33-7.12) | p= 0.048 |
| Amygdala Rt | 4.76 (-0.13-7.78) | 8.51 (5.30-9.63) | p= 0.322 |
| Cortex Lt | 6.14 (0.26-8.60) | 8.71 (7.95-11.54) | p= 1.273 |
| Cortex Rt | 6.38 (0.28-8.53) | 10.14 (6.01-11.07) | p= 0.258 |
| Hippo_C1&C2&C3&DG Lt | 9.42 (2.82-14.11) | 23.36 (18.53-33.04) | p= 0.011 |
| Hippo_C1&C2&C3&DG Rt | 12.90 (4.95-15.92) | 25.48 (16.33-28.47) | p= 0.024 |
| Hippo_C3 Lt | 8.18 (2.10-11.86) | 16.89 (14.66-20.99) | p= 0.024 |
| Hippo_C3 Rt | 8.59 (1.65-14.73) | 17.89 (10.64-21.93) | p= 0.120 |
| Thalamus Lt | 4.48 (-1.85-9.64) | 9.38 (3.87-10.09) | p= 0.396 |
| Thalamus Rt | 8.07 (0.68-8.83) | 11.99 (8.62-13.03) | p= 0.066 |
| **Post 10min** | | | |
| Amygdala Lt | 3.52 (0.45-6.27) | 7.24 (6.69-9.06) | p= 0.120 |
| Amygdala Rt | 5.48 (-2.50-8.48) | 7.96 (7.55-9.66) | p= 0.120 |
| Cortex Lt | 6.65 (-2.02-9.47) | 11.51 (9.38-12.53) | p= 0.120 |
| Cortex Rt | 7.31 (-2.02-9.31) | 10.25 (7.71-12.34) | p= 0.120 |
| Hippo_C1&C2&C3&DG Lt | 10.76 (-1.54-16.58) | 22.01 (16.26-30.83) | p= 0.048 |
| Hippo_C1&C2&C3&DG Rt | 13.27 (2.11-17.07) | 24.60 (17.33-30.07) | p= 0.024 |
| Hippo_C3 Lt | 7.84 (1.77-9.96) | 15.94 (13.53-24.98) | p= 0.016 |
| Hippo_C3 Rt | 8.49 (1.06-13.03) | 17.07 (15.69-20.99) | p= 0.120 |
| Thalamus Lt | 7.09 (-1.12-9.50) | 10.88 (9.02-11.46) | p= 0.090 |
| Thalamus Rt | 6.48 (-1.89-8.95) | 11.59 (10.04-13.51) | p= 0.034 |
| **Post 15min** | | | |
| Amygdala Lt | 4.02 (1.05-7.66) | 8.38 (6.28-10.78) | p= 0.090 |
| Amygdala Rt | 6.01 (-1.46-8.39) | 7.30 (6.54-11.47) | p= 0.322 |
| Cortex Lt | 6.78 (-1.13-9.52) | 9.23 (7.91-11.55) | p= 0.157 |
| Cortex Rt | 8.34 (-0.97-10.04) | 10.37 (9.12-12.11) | p= 0.120 |
| Hippo_C1&C2&C3&DG Lt | 11.27 (-2.39-17.60) | 20.34 (15.80-26.29) | p= 0.066 |
| Hippo_C1&C2&C3&DG Rt | 14.08 (2.10-19.69) | 22.43 (13.10-24.66) | p= 0.090 |
| Hippo_C3 Lt | 9.47 (1.75-12.00) | 18.03 (12.40-22.05) | p= 0.048 |
| Hippo_C3 Rt | 10.35 (1.53-13.57) | 15.96 (13.92-20.83) | p= 0.066 |
| Thalamus Lt | 8.85 (0.36-10.66) | 10.49 (8.71-13.79) | p= 0.322 |
| Thalamus Rt | 6.81 (-0.83-8.90) | 11.19 (9.44-13.38) | p= 0.066 |
| **Post 20min** | | | |
| Amygdala Lt | 3.90 (0.39-5.24) | 8.15 (6.92-11.60) | p= 0.016 |
| Amygdala Rt | 5.82 (-1.83-7.67) | 7.49 (6.79-12.70) | p= 0.258 |
| Cortex Lt | 5.84 (-1.24-9.59) | 10.85 (7.75-13.72) | p= 0.090 |
| Cortex Rt | 7.55 (-2.14-11.07) | 10.04 (8.68-12.32) | p= 0.479 |
| Hippo_C1&C2&C3&DG Lt | 9.67 (-2.76-18.09) | 18.68 (14.01-25.62) | p= 0.120 |
| Hippo_C1&C2&C3&DG Rt | 13.26 (1.66-18.78) | 23.37 (18.31-25.76) | p= 0.034 |
| Hippo_C3 Lt | 10.21 (3.28-13.21) | 16.43 (13.55-28.48) | p= 0.066 |
| Hippo_C3 Rt | 9.20 (0.87-14.38) | 17.29 (13.84-21.37) | p= 0.090 |
| Thalamus Lt | 5.71 (-1.37-10.45) | 11.21 (9.21-16.39) | p= 0.157 |
| Thalamus Rt | 8.07 (-2.05-10.22) | 11.47 (9.94-13.07) | p=0.090 |
| **Post 25min** | | | |
| Amygdala Lt | 4.77 (0.06-7.37) | 8.44 (5.06-10.51) | p= 0.203 |
| Amygdala Rt | 7.25 (-0.20-8.60) | 7.81 (4.54-13.96) | p= 0.396 |
| Cortex Lt | 6.99 (-1.62-8.63) | 10.25 (6.75-13.95) | p= 0.203 |
| Cortex Rt | 8.74 (-1.95-11.21) | 9.43 (7.71-13.53) | p= 0.480 |
| Hippo_C1&C2&C3&DG Lt | 11.28 (-0.21-17.14) | 19.41 (14.30-26.08) | p= 0.090 |
| Hippo_C1&C2&C3&DG Rt | 13.64 (2.05-18.79) | 19.36 (15.79-24.73) | p= 0.157 |
| Hippo_C3 Lt | 9.35 (2.02-11.61) | 15.31 (14.09-20.90) | p= 0.024 |
| Hippo_C3 Rt | 10.64 (2.83-13.47) | 15.22 (13.80-17.88) | p=0.090 |
| Thalamus Lt | 6.53 (-1.82-8.85) | 10.90 (7.87-14.91) | p= 0.048 |
| Thalamus Rt | 8.73 (-0.28-11.17) | 10.96 (8.94-12.77) | p= 0.258 |

Data list median (25th -75th percentile) of the signal enhancement calculated by (Smin-Spre)*100%/ Spre, where Smin is the signal at the scanned times of 5, 10, 15, 20, and 25min and Spre is the signal acquired before injection of the contrast media.

*P-value by Mann-Whitney test

**Supplementary Table S5. Results of the comparison of the signal enhancement among the scan time points for each mouse model.**

| **ROI** | **Post5min (1)** | **Post10min (2)** | **Post15min (3)** | **Post20min (4)** | **Post25min (5)** | ***F/p** |
| --- | --- | --- | --- | --- | --- | --- |
| **Control mice** | | | | | | |
| Amygdala Lt | 4.69 (3.30-8.44) | 6.25 (3.04-9.64) | 6.77 (3.04-9.69) | 5.24 (2.82-9.35) | 7.37 (3.82-10.12) | F= 1.090/P= 0.376 |
| Amygdala Rt | 6.30 (4.34-8.41) | 7.64 (1.18-8.52) | 8.01 (4.66-10.09) | 7.67 (1.03-9.35) | 8.60 (4.02-10.65) | F= 2.421/P= 0.066 |
| Cortex Lt | 6.89 (3.70-10.56) | 7.50 (5.12-11.27) | 7.57 (4.89-10.37) | 7.55 (4.06-11.57) | 7.18 (5.50-10.02) | F= 1.067/P= 0.387 |
| Cortex Rt | 7.39 (3.24-10.17) | 8.96 (3.34-9.96) | 9.19 (5.05-10.18) | 8.59 (4.65-11.15) | 8.76 (2.50-11.00) | F= 0.956/P= 0.443 |
| Hippo_C1&C2&C3&DG Lt | 16.05 (9.08-26.65) | 16.85 (5.25-23.15) | 16.31 (5.70-19.85) | 16.19 (4.16-21.00) | 16.29 (6.16-18.43) | F= 2.364/P= 0.071 |
| Hippo_C1&C2&C3&DG Rt | 15.92 (10.15-24.27) | 17.07 (9.98-21.69) | 18.22 (9.34-22.30) | 17.26 (9.49-20.71) | 17.23 (9.90-20.19) | F= 1.298/P= 0.289 |
| Hippo_C3 Lt | 10.33 (7.98-14.67) | 9.90 (7.41-12.87) | 11.00 (7.97-13.13) | 11.05 (7.33-15.19) | 9.96 (9.17-14.40) | F= 1.896/P= 0.132 |
| Hippo_C3 Rt | 10.91 (6.90-16.70) | 11.26 (8.19-17.28) | 11.42 (9.13-16.55) | 11.00 (7.83-18.18) | 11.25 (9.24-16.30) | F= 0.740/P= 0.571 |
| Thalamus Lt | 6.98 (3.12-10.77) | 8.14 (5.77-11.09) | 9.87 (5.88-11.17) | 7.28 (4.71-11.72) | 8.76 (4.94-9.86) | F= 0.868/P= 0.492 |
| Thalamus Rt | 8.24 (5.33-11.21) | 7.84 (5.47-10.42) | 7.46 (6.19-10.81) | 9.49 (6.15-11.16) | 9.94 (7.37-12.60) | F= 0.657/P= 0.626 |
| **3xTg mice** | | | | | | |
| Amygdala Lt | 6.03 (5.29-6.85) | 7.99 (7.31-9.88) | 7.76 (7.12-10.97) | 9.88 (7.00-12.62) | 9.77 (5.18-11.87) | F= 1.586/P= 0.202 |
| Amygdala Rt | 6.46 (5.71-8.74) | 9.66 (7.83-11.26) | 11.92 (6.91-13.00) | 12.18 (7.64-13.55) | 12.93 (4.23-14.44) | F= 1.179/P= 0.339 |
| Cortex Lt | 10.92 (9.40-13.07) | 12.55 (9.76-14.45) | 11.55 (8.55-16.25) | 12.00 (8.43-19.13) | 14.45 (7.38-18.35) | F= 0.394/P= 0.812 |
| Cortex Rt | 9.89 (7.96-11.11) | 11.50 (8.75-13.64) | 12.11 (9.09-15.47) | 11.62 (8.58-17.86) | 14.29 (7.05-19.43) | F= 0.394/P= 0.812 |
| Hippo_C1&C2&C3&DG Lt | 33.04 (25.49-47.55) | 33.08 (26.85-60.68) | 32.02 (24.72-71.91) | 31.31 (19.83-74.15) | 26.74 (18.67-76.26) | F= 0.416/P= 0.796 |
| Hippo_C1&C2&C3&DG Rt | 37.47 (23.84-51.00) | 36.54 (25.31-68.54) | 36.97 (21.51-74.23) | 36.58 (21.72-77.71) | 30.16 (16.98-77.01) | F= 0.330/P= 0.856 |
| Hippo_C3 Lt | 27.48 (16.93-40.73) | 30.47 (15.58-49.22) | 27.40 (17.04-53.85) | 28.48 (14.27-57.00) | 23.95 (13.52-57.29) | F= 0.594/P= 0.669 |
| Hippo_C3 Rt | 21.93 (15.75-34.12) | 21.58 (15.36-49.03) | 21.99 (12.07-55.87) | 21.37 (12.29-62.15) | 21.21 (13.92-65.48) | f= 0.182/p= 0.946 |
| Thalamus Lt | 10.05 (7.67-12.07) | 11.55 (8.89-16.82) | 13.87 (10.72-21.76) | 16.39 (9.04-27.43) | 14.91 (10.07-29.76) | F= 4.366/P= 0.006  (p= 0.055, 1,4)  (p= 0.039, 1,5)  (p= 0.008, 2,3)  (p= 0.020, 2,4)  (p= 0.012, 2,5) |
| Thalamus Rt | 11.22 (8.33-12.00) | 11.69 (11.13-16.22) | 13.57 (10.27-23.98) | 11.66 (9.67-26.89) | 12.77 (8.75-30.98) | F= 0.757/P= 0.561 |
| **APP/PS/ Tau** | | | | | | |
| Amygdala Lt | 4.84 (2.31-7.07) | 6.33 (4.53-8.54) | 8.23 (4.74-10.59) | 6.92 (4.10-9.26) | 9.16 (4.50-10.21) | F= 2.763/P= 0.040  (p= 0.032, 1,3)  (p= 0.123, 1,4)  (p= 0.102, 1,5) |
| Amygdala Rt | 5.65 (3.46-8.73) | 6.89 (4.13-9.18) | 7.68 (4.36-9.54) | 6.79 (3.534-7.478) | 8.86 (4.19-10.21) | F= 0.431/P= 0.785 |
| Cortex Lt | 8.90 (7.06-12.69) | 11.03 (9.95-12.39) | 9.68 (7.98-12.88) | 10.12 (5.76-13.86) | 8.66 (6.36-13.76) | F= 0.117/P= 0.976 |
| Cortex Rt | 9.41 (6.51-12.29) | 9.54 (7.25-12.14) | 10.48 (7.93-12.34) | 9.88 (5.55-11.88) | 9.89 (7.82-13.18) | F= 0.395/P= 0.811 |
| Hippo_C1&C2&C3&DG Lt | 21.77 (18.79-42.60) | 25.50 (17.27-45.21) | 19.99 (16.14-42.31) | 25.62 (16.25-38.06) | 21.15 (16.24-33.61) | F= 3.444/P= 0.016  (p= 0.278, 1,4)  (p= 0.123, 1,5) |
| Hippo_C1&C2&C3&DG Rt | 28.47 (19.12-47.02) | 29.03 (17.39-60.91) | 24.66 (13.61-54.48) | 25.76 (17.48-45.05) | 23.59 (16.52-42.51) | F= 4.508/P= 0.004  (p= 0.465, 1,5)  (p= 0.003, 2,3)  (p= 0.067, 2,4)  (p= 0.019, 2,5) |
| Hippo_C3 Lt | 20.99 (14.11-43.21) | 19.00 (13.91-49.82) | 18.30 (12.80-49.55) | 29.96 (14.06-41.28) | 16.54 (14.19-37.90) | F= 1.748/P= 0.159 |
| Hippo_C3 Rt | 18.44 (15.80-38.90) | 17.96 (13.08-41.42) | 16.83 (14.18-40.64) | 18.16 (13.88-37.87) | 16.32 (13.71-37.68) | F= 4.474/P= 0.004  (p= 0.413, 1,5)  (p= 0.067, 2,4)  (p= 0.024, 2,5) |
| Thalamus Lt | 10.00 (4.62-11.55) | 11.30 (9.49-14.02) | 9.31 (6.75-14.16) | 11.51 (8.24-18.63) | 11.00 (7.49-19.21) | F= 2.200/P=0.087 |
| Thalamus Rt | 12.38 (9.24-12.98) | 12.39 (11.12-13.54) | 13.05 (9.84-13.65) | 12.83 (10.42-17.38) | 12.79 (9.31-17.49) | F= 0.272/P= 0.895 |

Data list median (25^th^ -75^th^ percentile) of the signal enhancement calculated by (S_min_-S_pre_)*100%/ S_pre_, where S_min_ is the signal at the scanned times of 5, 10, 15, 20, and 25min and S_pre_ is the signal acquired before injection of the contrast media.

*P-value by Friedman test with F and p-values with the post-hoc analysis using Wilcoxon signed rank test after Friedman test

Abbreviations: DG, Dentate gyrus; Lt, left; Rt, right; ROI, region of interest

**Supplementary Materials: Cytotoxicity and Stability**

**Cell Culture***:* Even though these results may not accurately predict the in-vivo toxicity, it provides a basis for understanding the mechanism of cytotoxicity of DOTAGd-ob5 uptake at the cellular level. The bEND3 cells were derived from endothelial cells isolated from brain tissue derived from a mouse with endothelioma from BALB/c mice (American Type Culture Collection, Manassas, VA, USA; Catalog no. CRL-2299). The Cells were cultured in Dulbecco’s Modified Eagle’s Medium (American Type Culture Collection, Manassas, VA, USA) supplemented with 10% fetal bovine serum (R&D systems, Minneapolis, MN, USA), and 100 μg/mL penicillin/streptomycin (Sigma-Aldrich, St. Louis, MO, USA) at standard cell culture conditions at 37 °C in a 5% CO_2_ atmosphere.

**Microculture tetrazolium test (MTT assay)*:*** The inhibitory effect of DOTA-Gd-ob5, and proliferation of bEND3 and HT22 cell lines were assessed by up-taking of thiazolyl blue tetrazolium bromide (MTT, Sigma) by viable cells (*Mosmann T. Rapid colorimetric assay for cellular growth and survival: application to proliferation and cytotoxicity assays. J. Immunol. Methods .1983;65:55–63*). Both cells were plated onto 96-well plates (SPL, Pochen, KR) at a density of 2 × 10^3^ cell/100 µL/well. After incubation at 37 °C for 24 h, the medium was replaced with either control medium or medium containing specialized concentration DOTA-Gd-ob5 for 24, 48, 72 and 96 h. The concentration of DOTA-Gd-ob5 for cell culture treatment was 0.25, 0.5, 1, 2 and 5 mM, respectively and the concentration of zero means negative control. One hundred microliter of MTT solution (0.5 mg/mL) was added to each well and then the cells were incubated at 37 °C for 3 h. Following solubilizing the precipitated formazan with 100 µL DMSO, the optical densitometry was measured at a wavelength of 540 nm. The inhibition rate (IR) was evaluated using the following equation: IR (%) = 1 - OD_exp_/OD_con_× 100, where OD_exp_ and OD_con_ are the optical densitometries of treated and untreated cells, respectively. The viability rate of DOTA-Gd-ob5 was evaluated using the following equation: Viability (%) = 100 - IR (%).

**Cell Morphology**: The general morphology of the cell incubated with nanoparticles in phase-contrast microscopy is shown in Figure 2. At the end of 24 h, the exposed cells were washed with PBS and the cells were visualized by phase-contrast microscopy.


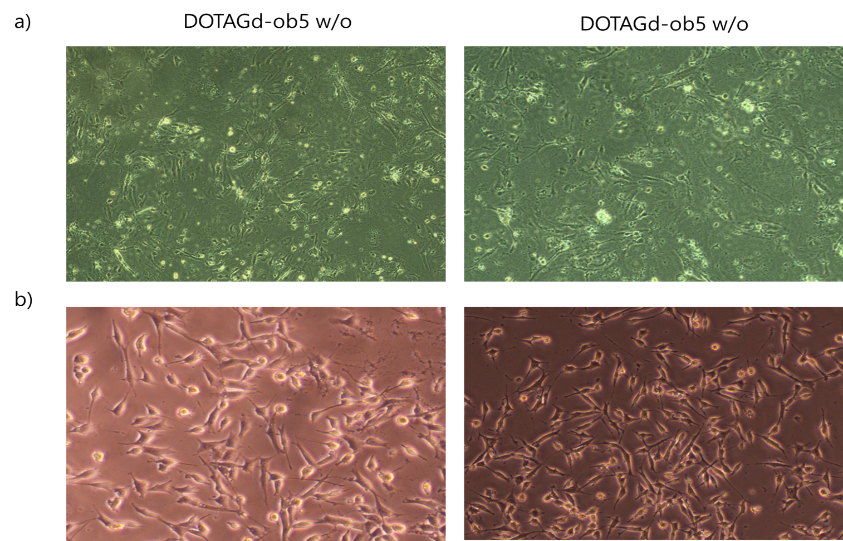


**Supplementary Fig S1**. Morphological characterization of the cells. Cells were treated with 1 mM concentrations of DOTAGd-ob5 in DMEM and incubated for 24 h at 37 °C in a 5% CO2 atmosphere. HT22 and bEND3 cell lines after incubation with DOTAGd-ob5 for 24 h. (a) HT22 cell lines without (left) or with DOTAGd-ob5 (right) (b) bEND3 cell lines after incubation without (left) or with (right) DOTAGd-ob5 for 24 h.

This figure shows bEND3 and HT22 cells that were well spread after incubation with 1 mM concentrations of DOTAGd-ob5 for 24 h, and there was no distinct change in morphology after incubation for 24 h with 1 mM DOTAGd-ob5 relatives. Similar results were obtained with DOTAGd only.


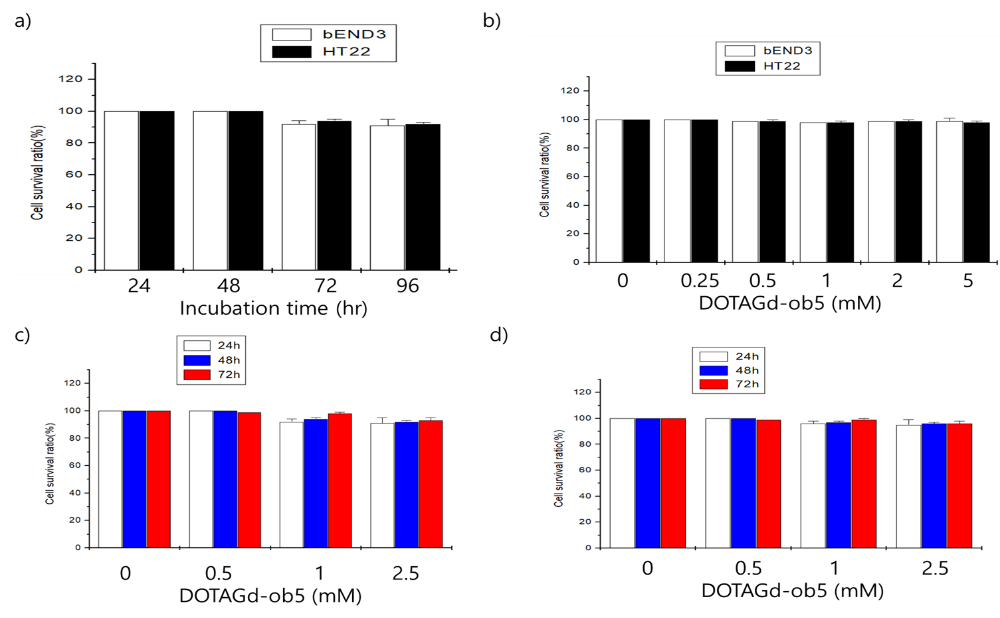


**Supplementary Fig S2**. Effect of DOTA-Gd-ob5 on cell survival ratio in bEND3 and HT22 cells. (a) incubation time, (b) different concentrations of DOTA-Gd-ob5 (c) different concentrations of DOTA-Gd-ob5 for 24, 48, and 72 h incubation time in bEND3 cells (d) different concentrations of DOTA-Gd-ob5 for 24, 48 and 72 h incubation time in HT22 cells. The data are expressed as mean ± SD of three independent experiments

The cell survival ratio of the cells was measured using the MTT assay after culturing in the presence of the DOTA-Gd-ob5 for 24 h. As is evident from [Fig. S2](https://www.ncbi.nlm.nih.gov/pmc/articles/PMC7059077/figure/F2/)a and S2b, the DOTA-Gd-ob5 had no significant cytotoxicity effect. bEND3 and HT22 cell lines were exposed to different doses of DOTA-Gd-ob5 for 24, 48, and 72 h as indicated in the Materials and Methods (Fig S2c and S2d). Each point represents a mean value and standard deviation of 3 experiments. Cell Viability in different concentrations is not significantly different (P < 0.05) compared to the control, as is evident from [Fig S2d](https://www.ncbi.nlm.nih.gov/pmc/articles/PMC7059077/figure/F3/). The MTT assay showed that DOTA-Gd-ob5 is less toxic than other DOTA-Gd (data not shown). The results of the MTT assay showed that DOTA-Gd-ob5 did not produce cytotoxicity in different concentrations. There were no significant differences with DOTA-Gd-ob5 being used as commercial contrast agents in Radiology.


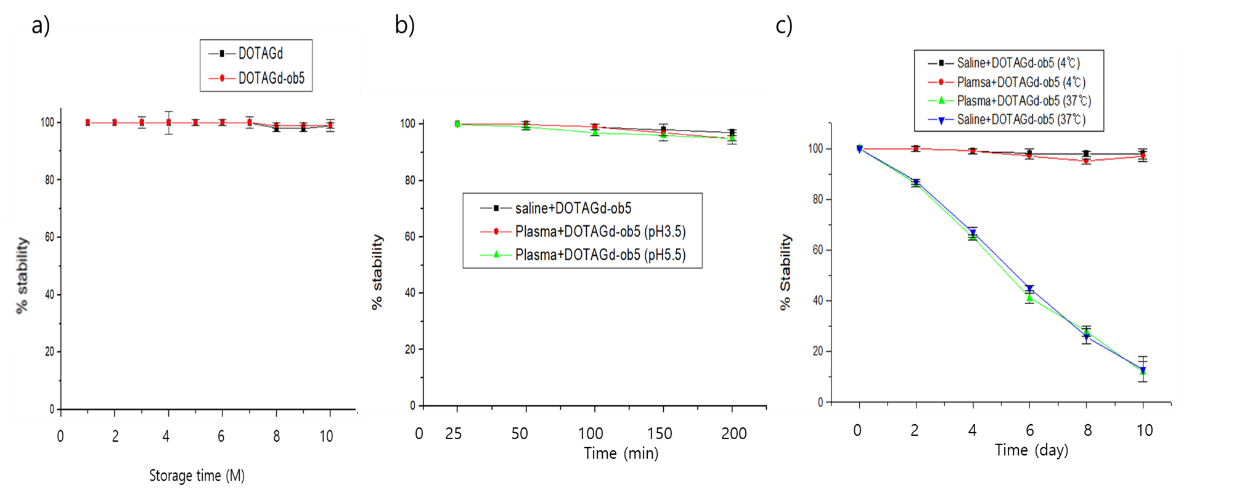


**Supplementary Fig S3**. In vitro DOTA-Gd-ob5’ stability of storage time human plasma at 37 °C and pH.

Small aliquots were analyzed by HPLC (a) 2-4-6-8-10 M for DOTA-Gd-ob5 and (b) pH 3.5 and pH5.5 for 0-25-50-100-150-200 min, and (c) 0-2-4-6-8-10 days for DOTA-Gd-ob5 at 4℃ and 37℃. The co-elution experiments showed that all the DOTA-Gd-ob5 described in this work retained the ability to form stable complexes with DOTA-Gd-ob5 aptamer (Fig S3).

DOTA-Gd-ob5 aptamer-conjugates were highly stable (>99% of intact compound) in 0.9% saline solution (black) and human plasma (red) at 37℃ for 10m. On the other hand, a stability test of the DOTA-Gd-ob5 conjugated batch showed the presence of lysis of about 2% every 24 h, which was significantly reduced to about 2% within 200 mins (Fig3b) and which was significantly reduced to about 89% within 10 days by 37℃ (Fig S3b). All the conjugated preparations of DOTAGd exhibited high hydrophilicity.
